# Insects as Sentinels of Oxidative Stress Induced by Environmental Contaminants: Biomarkers and Analytical Approaches

**DOI:** 10.3390/toxics13080698

**Published:** 2025-08-20

**Authors:** Marcello Messi, Roberta Giorgione, Maria Luisa Astolfi

**Affiliations:** 1 Department of Chemistry, Sapienza University of Rome, Piazzale Aldo Moro 5, 00185 Rome, Italy; marcello.messi@uniroma1.it (M.M.); roberta.giorgione@uniroma1.it (R.G.); 2 Research Center for Applied Sciences to the Safeguard of Environment and Cultural Heritage (CIABC), Sapienza University of Rome, Piazzale Aldo Moro 5, 00185 Rome, Italy

**Keywords:** biomarkers, enzyme activity, insects, low-molecular-weight antioxidant molecules, oxidative damage, oxidative stress

## Abstract

Despite their crucial biological role as metabolites, reactive oxygen and reactive nitrogen species (ROS and RNS) can have a negative effect on organisms when their cellular contents overwhelm the normal equilibrium provided by antioxidant defenses. Important biomolecules, such as lipids, proteins, and nucleic acids (i.e., DNA), can be damaged by their oxidative effects, resulting in malfunction or a shorter lifespan of cells and, eventually, of the whole organism. Oxidative stress can be defined as the consequence of an imbalance of pro-oxidants and antioxidants due to external stress sources (e.g., exposure to xenobiotics, UV radiation, or thermic stress). It can be evaluated by monitoring specific biomarkers to determine the state of health of breathing organisms. Assessments of ROS, RNS, specific degenerative oxidative reaction products, and antioxidant system efficiency (antioxidant enzyme activities and antioxidant compound contents) have been extensively performed for this purpose. A wide variety of analytical methods for measuring these biomarkers exist in the literature; most of these methods involve indirect determination via spectrophotometric and spectrofluorometric techniques. This review reports a collection of studies from the last decade regarding contaminant-induced oxidative stress in insects, with a brief description of the analytical methods utilized.

## 1. Introduction

With more than one million known species, insects represent the earth’s most prosperous group of animals. Insects have been popular as model organisms for more than 100 years for studying the themes of biology, such as aging and senescence [1,2], or diseases, such as dementia [3]. Over the past two decades, insects have emerged as bioindicators of environmental status [4,5,6,7,8,9]. As bioindicators, bees, mosquitoes, beetles, dragonflies, and moths can be used to measure the effects of environmental contaminants, such as heavy metals and pesticides, on ecosystems [4,9,10,11], and their protection is essential to ensure their continued use as models for the preservation of other living organisms, including humans. The use of insects as model organisms in oxidative stress studies is essentially linked to their ability to show rapid changes in morphological and physiological parameters, such as lifespan, brood size, and growth rate, and to the ease of management, which allows for the evaluation of exposure to chemical contaminants both in laboratory conditions and in real environmental conditions [8,12].

The toxic effects of contaminants on organisms can often be mediated by oxidative stress mechanisms, which may lead to sublethal outcomes [12,13,14,15,16,17,18,19,20,21]. Oxidative stress is generally defined as a detrimental condition resulting from an imbalance in the cellular oxidant/antioxidant system, in favor of oxidant species [22,23]. Within this framework, three main types of biomarkers are commonly used for assessment: (i) reactive species, either total or specific (RS); (ii) products of oxidative damage to biomolecules, such as lipids, proteins, or DNA; and (iii) components of the antioxidant defense system, including enzyme activities and low-molecular-weight antioxidants [14]. This classification clarifies the conceptual distinction between oxidative stress—representing the imbalance itself—and oxidative damage, which refers to the measurable biological consequences of that imbalance on cellular components. Reactive oxygen species (ROS) and reactive nitrogen species (RNS) are the primary classes of compounds (oxidant species) involved in oxidative stress. Even though they are generally referred to as free radicals because most of them are radicals (e.g., hydroxyl, ·OH; superoxide, O_2_·^−^; or nitrogen oxide, NO), they comprise a variety of nonradical RS that are able to produce an oxidative effect on the organism, such as ozone, singlet oxygen, H_2_O_2_, or nitrous acid [24]. Reactive oxygen species and RNS can be of both exogenous and endogenous origin: they can be generated by high-energy irradiation (UV light, X-rays, or gamma-rays); be products of metal-catalyzed reactions; be present as pollutants in the atmosphere; be produced by neutrophils and macrophages during inflammation; and be byproducts of mitochondria-catalyzed electron transport reactions and other mechanisms [25]. Because of their biological role in healthy cells and tissues as specific regulatory molecules [26], ROS and RNS can be referred to as metabolites [27].

In healthy cells and tissues, free radicals, or, more generally, RS, are controlled by an antioxidant system that reacts to their excess content or reactivity via the upregulation of antioxidants and related enzymes [26]. Therefore, an efficient antioxidant system is essential for maintaining the physiological level of RS and preventing damage to biological molecules. The antioxidant defenses present in animal species, such as insects, are based on similar mechanisms that can be categorized into three main groups for their typology of action [28]: the first group contains all the mechanisms that act to prevent RS reactions with biomolecules, such as superoxide dismutase (SOD), catalase (CAT), and selenium-dependent glutathione peroxidase (GPx-I); the second comprises those mechanisms for terminating RS chain reactions free radical scavengers, mainly low-molecular-weight antioxidants, such as glutathione (GSH), vitamins C and E, and carotenoids, which act in this way; and finally, the third group includes all the mechanisms responsible for the elimination of RS effects, for example, by repairing damaged biomolecules. Enzymes such as selenium-independent glutathione peroxidase (GPx-II) [29], glutathione S-transferase (GST) [30], and 8-oxoguanine glycosylase (OGG1) [31] are dedicated to this work.

External sources of stress (such as exposure to xenobiotics and irradiation) can induce RS overproduction in cells (or an efficient reduction in antioxidant defenses) when oxidant reactions overwhelm the defense system efficiency so that enzymes and radical scavengers are not able to detoxify the cell, oxidative stress conditions are reached, and damage to important biomolecules such as lipids, proteins, and DNA occurs [32,33]. The biomolecules most susceptible to oxidative damage are lipids: peroxidation of polyunsaturated fatty acids promoted by ·OH can lead to the production of two typical aldehydes (recognized as markers of lipid oxidative decay), malondialdehyde and 4-hydroxynonenale, with the latter able to react with proteins, impairing their functions [33]. Reactive species can also react directly with proteins: side-chain oxidation and backbone fragmentation can lead to carbonyl function formation, with consequent activity reduction [34]. Since most RSs are produced during cellular respiration, mitochondrial DNA is highly susceptible to oxidation; 8-oxoguanine (8-oxoG), with the formation of DNA strand breaks, is one of the most representative products of oxidative damage to DNA [31].

The determination of oxidative stress levels can be achieved by measuring the content of specific biomarkers, generally through indirect colorimetric or fluorometric assays, in which the formation or depletion of a specific reactant or an enzyme substrate is measured. These chemical assays can be susceptible to interference from the organic matrix under analysis; therefore, a specific pretreatment of the samples is often necessary.

This review briefly reports a collection of analytical methods, biomarker analyses, and sample treatments for assessing oxidative stress in insects induced by environmental contaminants, as previously discussed in Messi [35]. Articles published in the last decade were selected, including some highly relevant ones published more than 10 years ago, resulting from a keyword search for ‘oxidative stress’, ‘insect’, and ‘environmental contaminants’ (on Scopus or PubMed).

## 2. Temporal Distribution and Contextual Issues of Papers

**Figure 1:**
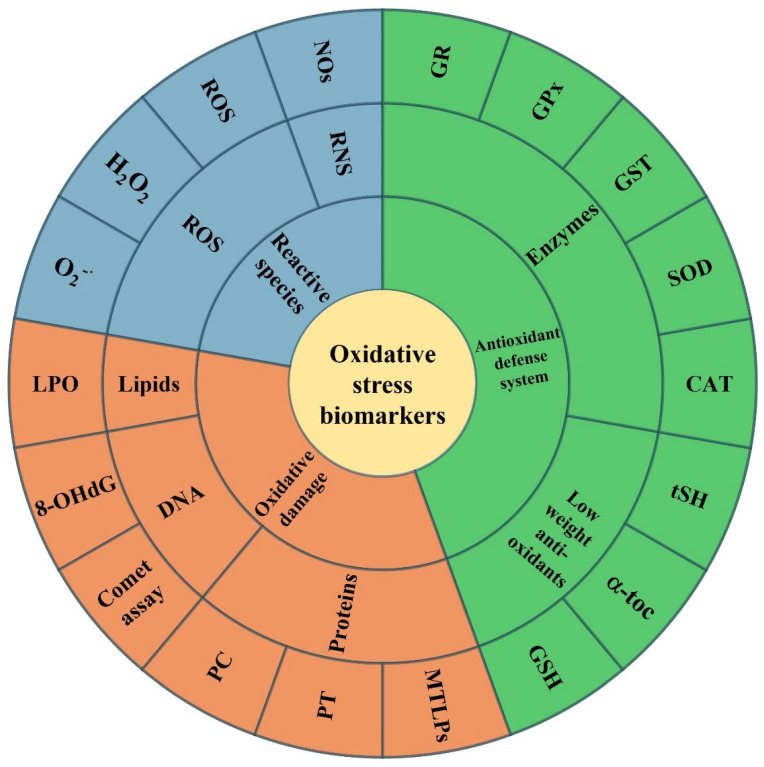
Biomarkers of oxidative stress are divided by typology: reactive species, the antioxidant defense system, and oxidative damage. 8-OHdG: 8-hydroxy-2′-deoxyguanosine; α-toc: alpha-tocopherol; CAT: catalase; GPx: glutathione peroxidase; GR: glutathione reductase; GSH: reduced glutathione; GST: glutathione-S-transferase; LPO: lipid peroxidation; MTLPs: metallothionein-like proteins; PC: protein carbonyl; PT: protein thiol; SOD: superoxide dismutase; tSH: total thiols.

Articles with at least one biomarker of oxidative stress were considered. Figure 1 visually categorizes the main biomarkers discussed in this review into three broad groups: RS, antioxidant defense systems, and markers of oxidative damage. This classification helps readers to better understand the different types of molecules involved in oxidative stress processes and provides a clear framework for interpreting the studies included in this review.

**Figure 2:**
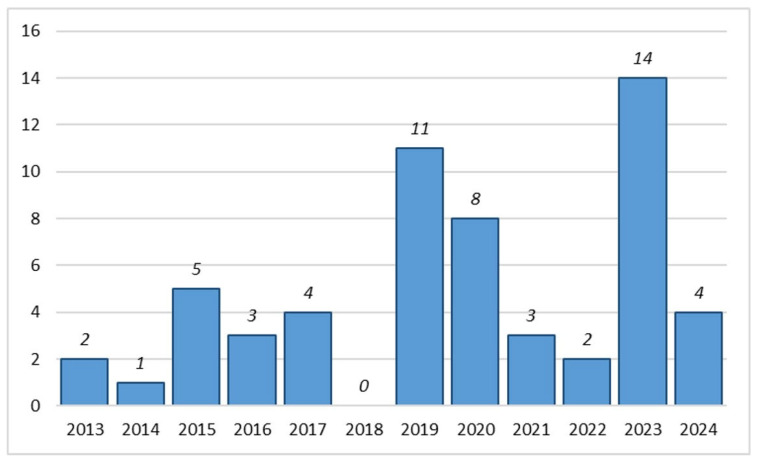
Number of articles by year.

**Figure 3:**
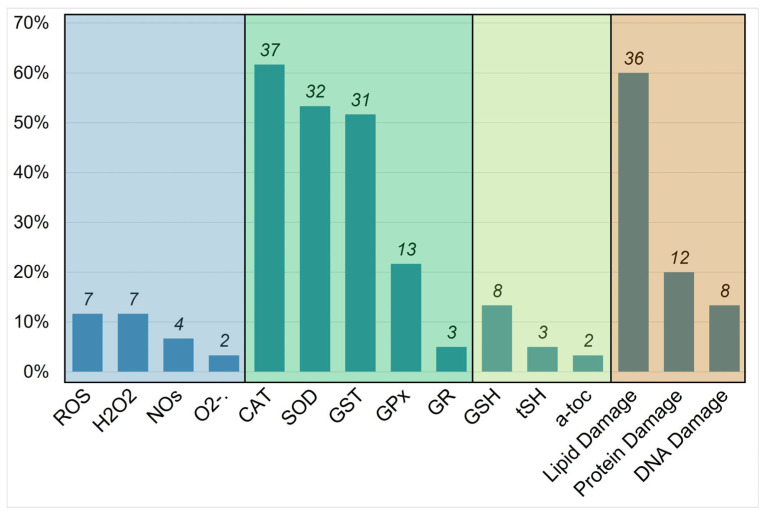
Occurrence of oxidative stress assays. Reactive species (blue), antioxidative enzyme activities (green), low-molecular-weight antioxidants (light green), and oxidative damage assays (orange).

**Figure 4:**
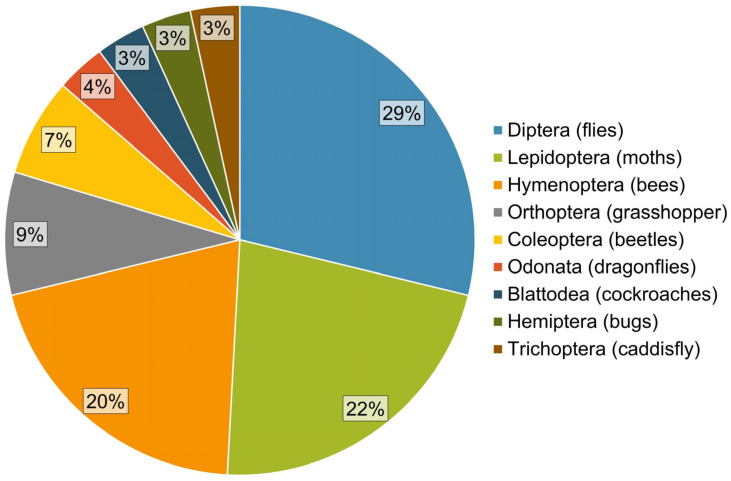
Occurrence of involved insects grouped by order; representative common names are reported in brackets.

The selection criteria limited the analysis to 57 articles (Figure 2), encompassing a total of 205 assays of oxidative stress (Figure 3), which were conducted on 27 different insect species belonging to 9 different orders (Figure 4). Further details on the methods considered—including insect species, sample type, exposure conditions, effects evaluated, analytical methods, and sample reading wavelengths—are provided in Table A1, Table A2, Table A3, Table A4, Table A5, Table A6, Table A7, Table A8, Table A9, Table A10, Table A11, Table A12, Table A13, Table A14 and Table A15.

Figure 2 shows the total number of articles by year. The year with the highest number of publications was 2023, with 14 articles (23%), and the year with the lowest number of publications was 2018, with zero articles. The distribution of papers over the years appeared well balanced, although with some fluctuations. Many studies have demonstrated that studying oxidative stress in insects is relevant and evolving.

Figure 3 shows the percentages of articles that used oxidative stress assays. Most articles used CAT and lipid damage assays (65 and 63% of articles, respectively), whereas 56 and 54% of the articles used SOD and GST assays. Catalase and SOD are antioxidant enzymes that are commonly studied together because they work simultaneously. In particular, SOD is a catalyst for the dismutation of O_2_^·−^ into H_2_O_2_, and CAT can eliminate H_2_O_2_, catalyzing the transfer of electrons [11]. As described in the following sections, CAT and SOD are very straightforward and practical tests that involve the evaluation of oxidative stress via UV-vis spectrophotometric analysis of the decrease in the absorbance signal of H_2_O_2_. Lipid damage may be associated with depletion of the antioxidant activity of SOD, CAT, and GST [36]. Methods using these latter assays will also be described in the following sections.

*Species* of interest in the reviewed articles are reported by *order* in Figure 4 as follows: *Periplaneta americana* [37], *Reticulitermes speratus* [38]), Coleoptera (*Anaceana globulus* [39], *Leptinotarsa decemlineata* [40], *Tenebrio molitor* [41], *Trachyderma hispida* [16]), Diptera (*Aedes aegypti* [42], *Chironomus kiiensis* [43], *Chironomus riparius* [20,44,45,46], *Drosophila melanogaster* [15,19,21,47,48,49,50,51,52], *Hermetia illucens* [53]), *Hemiptera* (*Oncopeltus fasciatus* [54,55]), *Hymenoptera* (*Apis cerana* [56], *Apis mellifera* [10,11,17,57,58,59,60,61,62,63], *Atta sexdens* [64]), *Lepidoptera* (*Bombyx mori* [65,66,67], *Galleria mellonella* [68,69,70,71,72,73], *Ostrinia nubilalis* [74], *Spodoptera exigua* [75,76], *Spodoptera litura* [77]), *Orthoptera* (*Acheta domesticus* [78], *Aiolopus thalassinus* [79,80], *Locusta migratoria* [13]), and *Trichoptera* (*Hydropsyche pellucidula* [81], *Stenopsyche marmorata* [82]). In addition, *Diptera*: *Chironomidae*; *Odonata*: *Gomphus* and *Lestes* [83]; and *Orthoptera*: *Acridoidea* [84] have also been studied.

As shown in Figure 4, the three most studied insects are flies (e.g., *D. melanogaster*), moths (e.g., *G. mellonella*), and bees (e.g., *A. mellifera*). This choice is likely because *D. melanogaster* and *G. mellonella* are economically important insects that are easy to breed, have numerous offspring, have a short development cycle, and have a known genome, whereas *A. mellifera* is a very valuable insect that needs to be protected. In addition, *D. melanogaster* exhibits behavioral and biochemical similarities with vertebrates. In particular, it shares a consolidated genetic homology with humans and, therefore, allows us to easily test alternatives that counteract oxidative stress-related disorders due to exposure to toxic substances in humans [18,47].

## 3. Analytical Methods

### 3.1. Sample Treatment

Typically, pooled samples or individual insects are washed with purified water, dried, weighed, and stored at −80 °C before analysis. The homogenization of samples is conducted on ice-chilled buffer [usually phosphate-buffered saline (PBS) or Tris-HCl at a specific pH, with EDTA, inhibitors of metabolic pathways, or other additives when required by the method or by necessity], water, a glass-Teflon grinder [42,48], a homogenizer [13,40], or a glass stick [85]. The homogenates are usually centrifuged at a low speed to separate the undissolved materials, and the supernatant is then collected and treated, depending on the method protocol. The nuclear, mitochondrial, and cytosolic fractions were collected via differential centrifugation. Hosamani and Muralidhara [48] reported that the nuclear fraction was obtained from the pellet after centrifugation at 7800× *g* for 10 min; then, the postnuclear supernatant collected was divided into mitochondrial and cytosolic fractions by centrifugation at 10,000× *g* for 10 min (the mitochondrial fraction as the pellet and the cytosolic fraction as the postmitochondrial supernatant). Different spinning conditions have also been reported: Tetreau et al. [42], according to Riaz et al. [86], separated the cytosolic fraction by ultracentrifugation at 100,000× *g* for 1 h at 4 °C; a nuclear fraction, especially that used in the comet assay, was reported to be collected by centrifuging at 2500× *g* for 5 min at 4 °C [16]. Further treatment steps can follow, and since they are linked to specific assay protocols, they will not be reported here for brevity (more information can be found in the methods references; a complete guide on insect hemolymph collection can be found in Łoś and Strachecka [87]).

### 3.2. Reactive Species Assays

#### 3.2.1. Nitrogen Oxides

The determination of nitrogen oxides (NOs) content in locusts [13] and fruit flies [15,47,49] was indirectly performed via colorimetric determination of nitrite (NO_2_^−^), and nitrate (NO_3_^−^) content via the Griess reaction (Table A1). The determination of NOs can be achieved by measuring the absorbance at 492 [88,89], 548 [90], or 550 nm [47,91] of the diazo-dye product of the two-step Griess reaction of dinitrogen trioxide (N_2_O_3_), generated by the acid-catalyzed formation of nitrous acid from NO_2_^−^ or by autoxidation of NO, with sulfanilamide and N-(1-napthyl)ethylenediamine (Griess reagent). When extracellular fluid quantification of NO_2_^−^ and NO_3_^−^ is needed, NO_2_^−^ reductase enzymes can be efficiently applied to reduce any NO_3_^−^ present to NO_2_^−^ before determination [88]. Grace insect medium, which is a modification of Wyatt’s medium, can support the maintenance of insect cells [90]. The samples were incubated at room temperature with Griess reagent at a 1:1 ratio [47,49] for 20 min [47,49]. The NO content is reported in NO_2_^−^ equivalents (nmol/L), referred to as the NO_2_^−^ calibration curve [47,49], and is finally normalized to grams of protein [13], with the advantage of easy comparison. Alternatively, NO content is expressed with respect to the weight of the tissue analyzed [15].

In fruit flies exposed to Cd(II) [47] in combination with Fe(II) + rotenone [49], an increased level of NO was detected. However, when exposed to rotenone at only 50 µM, an increase in NO content was observed when the fly’s whole body was analyzed [15], whereas no significant change was observed in the fly’s head [49]. An amelioration of NO levels, attributed to the antioxidant properties, was observed when Cd(II) and rotenone were administered to flies with the flavonoid hesperidin or *Syagrus coronata* fixed oil, respectively [15,49], reverting their values to the control levels.

#### 3.2.2. Reactive Oxygen Species

Reactive oxygen species (Table A2) were monitored in silkworms, termites, fruit flies, and beet armyworms via fluorescence emission of 2,7-dichlorofluorescein (DCF) resulting from 2,7-dichlorofluorescein diacetate (DCF-DA) oxidation [21,48,50,65,66,75]. In contrast, Peng et al. [41] reported the use of dihydroethidium (DHE) at an excitation wavelength (Ex) of 488–535 nm and an emission wavelength (Em) of 610 nm to determine the ROS content in mealworm larvae. The DCF method consists of adding DCF-DA to the sample and incubating it in the dark at room temperature [48] or 37 °C [50,65,75] for a specific interval from 15 min [48] to 1 h [92], depending on the author. After this period, the quantification of ROS is determined by comparison of fluorescence units (at Ex = 485–500 nm and Em = 525–530 nm) and expressed in relative fluorescence units with respect to a blank [50,65] or in pmol of DCF formed per minute [48]; standardization to protein weight was sometimes also adopted [48,65].

The exposure of female silkworm individuals to graphene oxide nanoparticles (GONPs) increased ROS levels in ovary tissues [66]. An increase in the concentration of ROS was also observed in fruit flies exposed to *Eugenia uniflora* leaf essence oil (used in popular medicine) [50] or the pesticide paraquat [48], as well as in fruit fly [21] and beet armyworm cell cultures [75] exposed to Sb(III) and camptothecin (alkaloid isolated from *Camptotheca acuminita*) [93], respectively. The midguts, Malpighian tubules, and fat body tissues of silkworm larvae fed polystyrene nanoparticles did not significantly differ from those of the control larvae [65].

#### 3.2.3. Hydrogen Peroxide

The methods for determining H_2_O_2_ in insects (honeybees, silkworms, fruit flies, and black soldier flies) are shown in Table A3. The H_2_O_2_ content in honeybees exposed to environmental pollution [10] or fed Se(IV) or Se(VI) [57] was determined with commercial kits. H_2_O_2_-specific oxidation of Amplex Red to resorufin (Abs: 570 nm; Ex/Em = 535/587 nm) was employed. Fang et al. [66] reported the same working wavelength, which used a commercial kit for the assay without providing enough information to retrieve the method.

Fruit flies exposed to Cd(II) [47], paraquat [48], or Fe(II) and rotenone [49] were analyzed according to Wolff’s method [94]. The colorimetric assay is based on the FOX 1 reagent, where the H_2_O_2_-specific oxidation of ferrous ions in xylenol orange (ferrous ammonium sulfate, sorbitol, sulfuric acid, and xylenol orange) is followed at 560 nm.

Finally, Abdelfattah and Renault [53] evaluated the H_2_O_2_ concentration in black soldier fly larvae fed different treated feeds (Cd, Fe, Pb, or catechol) via triiodide spectrophotometric detection [95]. The method is based on oxidation by H_2_O_2_ in an acidic medium of potassium iodide to form triiodide, which shows two intense absorption peaks at 285 and 350 nm. However, the reported working wavelength for triiodide determination is 240 nm.

A calibration curve of H_2_O_2_ was employed for quantification. The results are reported in various units: µmol/mL [47], µmol/L [49], nmol/mg of protein [48,66], nmol/mg of sample [10], ppm [53], or nmol/individual [57].

According to the authors, the H_2_O_2_ content significantly increased, with respect to the control, in honeybees exposed to Se(IV) or Se(VI) [57]; in silkworm ovarian tissues exposed to GOMPs [66]; in fruit flies exposed to Fe(II) and rotenone together, but not individually [49]; in those exposed to Cd(II) [47]; and in those exposed to paraquat [48]. An increase in H_2_O_2_, attributed to its antioxidant properties, was observed when Cd(II) was administered to *S. coronata* fixed oil-treated flies [47], which returned H_2_O_2_ to control levels.

#### 3.2.4. Superoxide

Specific determination of O_2_^−^∙ was performed on fruit flies [48] and mosquito larvae [42]. Two different analytical methods were used (Table A4). One is based on the specific oxidation of dihydroethidium (DHE) by superoxide anions into fluorescent 2-hydroxyethidium [96], with an optimal Ex/Em of 490/590 nm reported [48]. The other is based on the reduction of nitroblue tetrazolium (NBT) chloride by O_2_^−^∙ via a one-electron transfer reaction to yield monoformazan (NBT+) [96]. In accordance with Reynaud et al. [97], with some modifications, high specificity and precise quantification of superoxide concentration were reported by Tetreau et al. [42]. Briefly, the author added NBT directly to the sample (mosquito larvae) and incubated it for 2 h in darkness at room temperature. The nonreacted NBT was washed twice in ethanol, and the formazan (NBT+) formed was air-dried and then dissolved in a KOH/DMSO solution. A working wavelength of 630 nm was set to measure NBT+ absorbance. The results are reported for superoxide generated in mitochondria as “fluorescence units/min/mg of protein” [48] or directly as the optical density, OD_630_ [42].

In fruit flies exposed to paraquat (PQ) for 24 h, the superoxide content increased significantly for a PQ dose higher than 20 mM [48] compared with that of the control. To simulate low to high (0.5–50 µg/L) polycyclic aromatic hydrocarbon (PAH)-contaminated water exposure [42], mosquito larvae were treated with fluoranthene and/or benzo[a]pyrene (BaP) for 24 h and then irradiated with or without UV-A rays (365 nm) for 1 h (UV index = 1.14). The results indicated that superoxide formation was dependent on dose and treatment. An increase in PAH concentration led to a decrease in superoxide formation, especially when BaP was utilized. Irradiation treatment was observed to be responsible for a significant increase in superoxide content, which was 80% greater than that of the corresponding control. Considering the low degree of irradiation to which the larvae were exposed, this result highlights the importance of working in a controlled environment (e.g., in darkness) for the risk of alterations or artifacts.

### 3.3. Enzyme Activity Assays

#### 3.3.1. Catalase Activity

Oxidative stress conditions were evaluated by measuring CAT activity in insects of various typologies, mainly model organisms, exposed to various contaminants (Table A5). The activity of the CAT enzyme, which is responsible for the regulation of cellular peroxides, has been evaluated in *Orthoptera*, which are exposed to nanoparticles [13,78], insecticides [84], and different types of severe environmental pollution [79,80]; in termites, beetles, and bugs, which are exposed to ferrous ions [54], pesticides [37,40], and environmental pollution or irradiation [16,38,39]; in bees, which are exposed to heavy metals [56,58] and pesticides [59,60]; in *Lepidoptera*, which are exposed to nanoparticles [65,66,67,68,69], heavy metals [70], pesticides and plant growth regulating compounds [71,72,73,77]; and in flies, which are exposed to azo dyes [19], essential oils [50], pesticides [20,44,48,49], antibiotics [45], metals [47,49], and environmental pollution [46,82,83]. The choice of the proper insect is driven by the aim of the specific study; for example, terrestrial beetles were taken into consideration in soil-related studies [16], and aquatic insects were studied for water contaminants [39].

Commonly, the method utilized for measuring the enzyme activity of CAT consists of monitoring the consumption of H_2_O_2_ by a sample at 240 nm in potassium or sodium phosphate buffer (pH 7.0) [11,73,98,99,100,101,102,103,104,105,106,107,108,109] or Tris-HCl (pH 7.5) and EDTA [39,110] for a few minutes at 22, 25, or 37 °C. Rainio et al. [40], according to Deisseroth and Dounce [111] and Fossati et al. [112], reported the quantification of H_2_O_2_ using a different chromogenic system by the reaction of 3,5-dichloro-2-hydroxybenzenesulfonic acid (DCHBS) with 4-aminophenazone (ampyrone) and H_2_O_2_, catalyzed by horseradish peroxidase enzyme (HRP), with the formation of a chromophore product having a strong absorbance peak at 520 nm. Similarly, to shift the working wavelength from the UV to the visible range (405 nm), residual H_2_O_2_ was quantified by measuring the yellowish complex obtained by the addition of ammonium molybdate (a commercial kit used by Li et al. [56]). Like the previous method, this method consists of the indirect quantification of H_2_O_2_ decomposed at specific intervals by stopping enzymatic activity and measuring the depletion of the initial H_2_O_2_ via a secondary reaction.

Assessment of CAT activity via gel zymography was performed by Manna et al. [84] on field insects of *Acridoidea* (order *Orthoptera*), according to Zerbetto et al. [113]. Optical density comparison of zymogram bands allows the relative quantification of protease enzyme activities. ImageJ software was used for this purpose. The results were generally expressed as µmol H_2_O_2_ decomposed (or decreased in absorbance)/min/mg protein. The H_2_O_2_ concentration was determined from the measured absorbance using the Beer–Lambert law. Different values of the molar extinction coefficient at 240 nm have been reported in the literature (35.0 [37], 39.4 [51], 44.1 mM^−1^ cm^−1^ [48]), or alternatively by comparison with a standardized CAT solution [82].

Generally, the results obtained have led to a significant dependency on the dose or exposure time of the contaminants [45,60,69,73,77] in relation to the specific insect explored, e.g., gender-dependent [51], age and social role [59], and tissue analyzed [69,78]. A significant correlation between CAT activity and bioaccumulated metals (Fe, Mn, and Zn) was found in honeybees exposed to different types of environmental pollution [11].

#### 3.3.2. Superoxide Dismutase Activity

Oxidative stress conditions were evaluated in various insects, and the activity of the enzyme responsible for the dismutation of superoxide anions was monitored (Table A6). Both CAT and SOD have indeed been investigated in relation to H_2_O_2_ and superoxide anions, respectively, since they are considered the first antioxidant cellular response to these ROS.

According to the reviewed studies, SOD activity can be indirectly determined by monitoring a secondary chromogenic redox reaction, the mechanism of which is related to superoxide anions. Then, a competition mechanism is set, and the activity of the SOD enzyme can be evaluated by comparing the inhibition of the autoxidation rate of various well-known systems with respect to a reference (a control free of the SOD competition reaction). The systems used in the reviewed articles include pyrogallol [64,77,81,114,115], epinephrine [21,53,79,80,116], quercetin [48,50,51,117,118], and BXT-01050 (a tetracyclic catechol) [16,119] autoxidation. An alternative proposed mechanism relies on coupling the superoxide anion scavenging reaction (involving SOD) with a chromogenic redox reaction promoted by superoxide anion, which is produced in situ by the xanthine/xanthine oxidase system. For this purpose, NBT [19,120], p-iodonitrotetrazolium (INT) [59,121], tetrazolium salt (WST) [82,83], and cytochrome-C [44,45,65,68,69,70,122] oxidation can be monitored. This second methodology is preferably used in commercial kits [13,40,41,56,60,66,71,72,73,82] since ad hoc systems are easily made and branded by companies. An alternative to NBT that leads to water-insoluble mono- or diformazan, which needs to be solubilized in DMSO before the absorbance at 560 nm is read [19,120], is the water-soluble tetrazolium salt “WST-1” [82,83,123]. This salt overcomes some drawbacks of the use of NBT, mainly by leading to the formation of a water-soluble form of formazan at 450 nm and avoiding direct interactions with xanthine oxidase [123]. All these methods share a similar principle; essentially, they differ in the specific working conditions (e.g., pH and temperature) that depend on the optimal conditions for the compounds employed. In contrast, as previously reported for CAT determination, according to Weydert and Cullen [124], gel zymography techniques can be applied for the determination of SOD activity [84]; the support of image analyzer software and long analysis time must be considered.

As usual for enzymatic activity measurements, the results were reported in enzymatic units, defined as the concentration of SOD able to reduce the rate of reaction of the competitive system by 50%, which was monitored spectrophotometrically for a short interval at a specific wavelength (see Table A6) depending on the chromophore product formed.

Like CAT activity, SOD activity changes in a contaminant dose- and exposure time-dependent manner [60,69,71,72,77]. La Porta et al. [11] reported a significantly moderate to strong correlation between SOD activity levels and some bioaccumulated metals (Cr and Mn) in honeybees from different polluted environments.

#### 3.3.3. Glutathione S-Transferase Activity

Glutathione S-transferase catalyzes the conjugation of GSH with organic compounds through their electrophilic centers, initiating the detoxification process by neutralizing their alkylating potential and increasing their solubility in water; it is also responsible for GSH-mediated peroxide reduction [30]. The determination of GST activity was performed in *A. aegypti* [42], *A. mellifera* [11,59,60,61,62], *A. cerana* [56], *B. mori* [65], *C. kiiensis* [43], *C. riparius* [20,44,46], *D. melanogaster* [47,48,49,50,51,52], *G. mellonella* [68,69,70,71,72,73], *H. pellucidula* [81], *L. decemlineata* [40], *L. migratoria* [13], *O. fasciatus* [54], *S. litura* [77], and *T. hispida* [16] (Table A7), followed by a spectrophotometer at 340 nm to determine the formation of S-(2,4-dinitrophenyl)glutathione, the product of the conjugation of GSH with 1-chloro-2,4-dinitrobenzene (CDNB) [86,98,105,125,126,127,128,129,130,131,132]. The authors differ from each other by a few modifications in the protocol applications; generally, a sample aliquot was added to a reaction mixture made of GSH and CDNB in PBS at pH 6.5 [11,48,77], 6.9 [42], 7.0 [47,49,50,52], or 7.4 [61,62,65] in the presence or absence of EDTA and other additives, e.g., protease inhibitors (phenylmethylsulfonyl fluoride, PMSF), or dithiothreitol (to prevent GSH oxidation) [43]. The reactions were conducted at a constant temperature (ranging from 25 to 30 °C), and the absorbance was observed after a few minutes when the samples stabilized (from 1 to 8 min).

Glutathione S-transferase activity is usually reported as nmol of conjugated CDNB/min/mg protein through a calibration curve or by applying the Beer–Lambert equation with ε340 = 9.6 mM^−1^ cm^−1^ [54,73] or ε340 = 5.3 mM^−1^ cm^−1^ [46].

Similar to what has been previously reported for other enzyme activities (CAT and SOD), dependency on dose [52,59,60,61,71,72], time of exposure [61], age and social role [59], sex [51], and analyzed tissue [61] has been observed for GST activity changes. In addition, low-intensity UV-A irradiation (UV index = 1.17) significantly increased GST activity in mosquito larvae [42]. Therefore, all these are external factors that should be taken into consideration when comparing enzyme activities.

#### 3.3.4. Glutathione Reductase Activity

**Figure 5:**
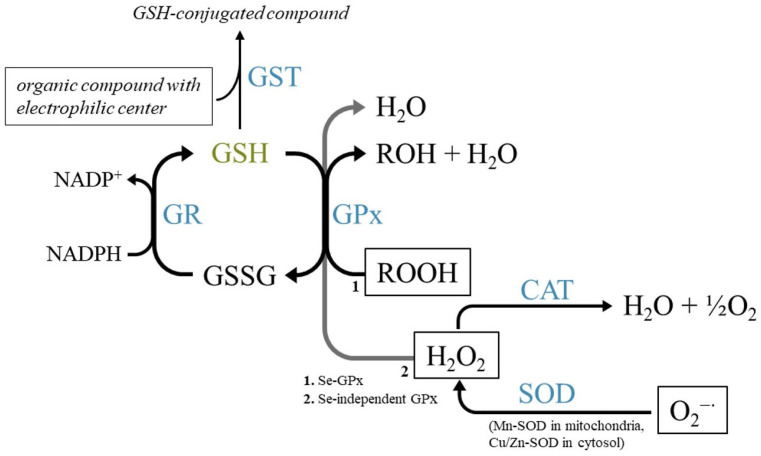
The biochemical environment of glutathione.

Assessment of glutathione reductase (GR) activity (Table A8) was performed to evaluate the regenerative capacity of glutathione in its reduced form (GSH), which is responsible for the functionality of GSH-related enzymes (such as GST and GPx) (Figure 5).

The traditional determination of GSH content is conducted via reaction with Ellman’s reagent (DTNB) [15,49,133], although this method involves thiol interference; therefore, the total glutathione content can be determined by monitoring the depletion of DTNB when an excess of GR and NADPH is present. Glutathione reductase activity can be determined by monitoring the formation of NADP+ when there is an excess of GSSG. When GPx activity is requested, the formation of NADP+ can be monitored while consuming a known quantity of organic hydroperoxide, with an excess of GR and NADPH; H_2_O_2_ can also be used as a substrate, but care must be taken to reduce competitive CAT activity (e.g., by adding sodium azide). Spectrophotometric determination of GR activity was performed by following the formation of GSH from a known quantity of the GR substrate oxidized glutathione (GSSG). Two approaches were followed: the first [126,134] consists of monitoring the formation of GSH by following the oxidation of NADPH to NADP+ associated with the process [11,44], where a decrease in absorbance is monitored at 340 nm, ε340 = 6.22 mM^−1^ cm^−1^ [126]; the second [40] is based on quantifying the GSH formed at various intervals by reaction with DTNB, of which the reduced form yields an increase in absorbance at 412 nm. Glutathione reductase activity was expressed in nmol of NADP+ produced/min/mg protein [44].

Glutathione reductase activity changes in samples of potato beetles exposed to glyphosate [40] and harlequin flies exposed to spinosad and indoxacarb [44] did not differ significantly from the reference. Instead, a moderately significant negative correlation was observed between GR activity levels and bioaccumulated Ni and Fe concentrations in honeybees; no significant relationship was reported with the other metals investigated [11].

#### 3.3.5. Glutathione Peroxidase Activity

The reviewed studies evaluated the cellular capacity of reducing hydroperoxide variations by comparing GPx activity between treated samples and reference samples in *A. domesticus* [78], *A. globulus* [39], *A. mellifera* [11,59], *C. riparius* [44], *D. melanogaster* [19], *G. mellonella* [68,69,70,72], *H. pellucidula* [81], *L. decemlineata* [40], and *L. migratoria* [13]. GPx exists in two main forms. The first, GPx-II, is able to catalyze the reduction of organic hydroperoxides to the corresponding alcohol and water; the other, GPx-I, which is selenium dependent, is responsible for the reduction of H_2_O_2_ [29,30,135] (Figure 5). In the reviewed articles (Table A9), the methods adopted for assessing GPx activity are based on the same mechanism described by Lawrence and Burk [135] and Paglia and Valentine [136], with the following modifications [137,138,139]: the assay consists of monitoring the oxidation of NADPH to NADP+ generated from the recycling reaction of GSSG to GSH, catalyzed by GR, and associated with the action of GPx on a hydroperoxide substrate. The most commonly employed substrates are cumene hydroperoxide [70,78] and H_2_O_2_ [11,40]. To prevent interference caused by CAT activity on the shared substrate [140], sodium azide (NaN_3_) was used when H_2_O_2_ was used.

Generally, the reactive mixture was prepared in EDTA and PBS, and the reaction was monitored at 340 nm. An extinction coefficient of 6.22 mM^−1^ cm^−1^ [72,126,141] was used. Glutathione peroxidase activity was determined by Paleolog et al. [59] through the purpurogallin test [100]. As described previously [87,105], a mixture of pyrogallol and H_2_O_2_ in PBS buffer at pH 6.8 was incubated for 5 min at 25 °C after the sample was added. Consequently, the reaction is stopped with H_2_SO_4_, and the formation of purpurogallin, resulting from the oxidation of pyrogallol, facilitated by peroxidase and H_2_O_2_, is determined with a spectrophotometer at 420 nm.

The quantification of GPx activity is expressed in terms of nmol of NADP+ produced/min/mg protein [44,68,69,72,81], units/mg protein [13,19,39,59,70], or µmol Cumene-OOH/min/mg protein [78].

### 3.4. Low-Molecular-Weight Antioxidant Molecule Assays

#### 3.4.1. Glutathione

Oxidative stress conditions can be evaluated through the glutathione content for its antioxidant and antitoxic behavior as a cofactor of GPx and GST [142,143]. This tripeptide (consisting of glycine, cysteine, and glutamic acid) plays a key role in the antioxidant defense by acting as a reductant against potentially toxic H_2_O_2_ and other peroxides, such as lipid hydroperoxides. Because of its nucleophilic behavior, it is also responsible for xenobiotic detoxification through a mechanism that is promoted by conjugation through the cysteine thiol group to the electrophilic centers (Figure 5). The levels of glutathione used for assessing changes in beetles or bugs exposed to heavy metal-polluted water [39] or pesticides [37,40] and ants [64] or flies exposed to colorant additives [19], pesticides [20,48], or Sb [21] are reported in Table A10. The principle of the assay is generally based on the reaction of GSH with Ellman’s reagent, 5,5′-dithiobis-(2-nitrobenzoic acid) (DTNB), to form glutathione disulfide (GSSG) and 2-nitro-5-mercaptobenzoic acid, the latter exhibiting an intense absorbance peak at 412 nm [133,144,145,146]. However, the application of this method produces a response for the total reduced thiols; to avoid interference from other thiols, a specific, sensitive, rapid, and reliable procedure for the total glutathione assay has been adopted [20,64]. The procedure follows the depletion of DTNB by the action of GSH, which is continuously recycled via the GR/NADPH system [147,148]. To reduce the contribution of protein thiols, trichloroacetic acid (TCA) [37] or sulfosalicylic acid (SSA) [40] was added to separate the proteins via precipitation. The estimation of the ratio of reduced to oxidized glutathione (GSH:GSSG), which is used as an index for oxidative stress assessment, was subsequently performed by Rainio et al. [40] via a commercial kit (K005-F1, Arbor). In this method, GSH is determined via a fluorescent probe (ThioStar^TM^, Ex/Em = 405/510 nm) as a substitute for DTNB in Ellman’s procedure; subsequently, the GSSG assay is conducted via the activation of the GR-NADPH recycling system. Total glutathione is then calculated by adding the contributions of GSH and GSSG [149].

Hosamani and Muralidhara [48] reported a different fluorometric procedure than previously described; the method consists of measuring the formation of a fluorescent complex, o-phthalaldehyde (OPT), with both reduced and oxidized glutathione at Ex/Em = 345/425 nm [150]. The addition of formaldehyde suppresses the interference of endogenous histidine-containing compounds. A calibration curve with GSH standards was used to quantify GSH via each method described.

#### 3.4.2. Total Reduced Thiols

The levels of total reduced thiols (nonenzymatic antioxidants), as shown in Table A11, were investigated in fruit flies exposed to Cd(II) [47] or rotenone pesticide [15,49]; in each study, the assessment of total reduced thiols (nonenzymatic antioxidants) was performed via Ellman’s reagent method [133,151]. The samples were prepared in phosphate buffer (pH 7.4), and the DTNB reagent was added and incubated at room temperature for 30 min [15,47,49]. The formation of a yellowish product (2-nitro-5-mercaptobenzoic acid) was then read at 412 nm [47,49,133]. A working wavelength of 405 nm was also reported [15].

The results were reported, referring to a GSH calibration curve, in µmol of GSH/mg of protein [15,47,49], or µmol of GSH/g of tissue [15]. They showed a significant decrease in RSH content in fruit flies exposed to rotenone, but only when the whole body was analyzed [15], or in heads when rotenone was administered together with FeSO_4_ [49].

#### 3.4.3. α-Tocopherol

Alpha-tocopherol (vitamin E) is a significant nonenzymatic antioxidant with the ability to counteract the oxidation of polyunsaturated fatty acids of biological membranes [152]; its change in content, relative to the control, has been studied in honeybees exposed to metals [17] or to various combinations of pesticides and metals [63] (Table A12). The assessment was conducted using the liquid chromatography technique, according to a method described by Helmer et al. [153], through hexane/acetone extraction and reversed-phase column separation (in a methanol/water gradient), with analytical detection at 292 nm [17,63].

Honeybees exposed for 10 days to environmentally relevant levels of Al(II), Cd(II), Pb(II), Fe(II), atrazine, and glyphosate revealed a concentration dependency of α-tocopherol content versus Cd and Pb [17]; nonsignificant variations were observed in the other contaminants investigated [17,63].

### 3.5. Oxidative Damage Assays

#### 3.5.1. Lipid Damage

Lipid peroxidation has been studied in *A. thalassinus* [79,80], *A. globulus* [39], *A. cerana* [56], *A. mellifera* [17,60,63], *B. mori* [65,66], *C. riparius* [20,44,45], *Chironomidae*, *Gomphus*, and *Lestes* [83], *D. melanogaster* [15,19,21,47,48,49,50,51], *G. mellonella* [72,73], *H. pellucidula* [81], *L. decemlineata* [40], *L. migratoria* [13], *O. fasciatus* [54,55], *O. nubilalis* [74], *P. americana* [37], *R. speratus* [38], *S. exigua* [75,76], *S. litura* [77], *S. marmorata* [82], *T. molitor* [41], and *T. hispida* [16] (Table A13). Oxidative stress can be assessed by measuring the levels of thiobarbituric acid reactive substances (TBARSs) as byproducts of lipid peroxidation (LPO), particularly malondialdehyde (MDA) [13,15,16,17,19,20,37,39,44,47,49,50,54,63,66,75,76,77,153,154,155,156,157,158,159,160,161,162]. Since the assay is not MDA-specific and a wide range of compounds (not related to LPO) also react with TBA [163,164], this method estimates TBARSs. Malondialdehyde or its precursors, such as 1,1,3,3-tetramethoxy-propane [155], are generally used as external standards to quantify TBARS content. Otherwise, a coefficient of molar extinction, ε535 = 1.56 × 10^5^ M^−1^ cm^−1^, is also used [37,72,73].

Another approach used for determining LPO consists of analyzing the lipid hydroperoxide (LHP) content obtained through the ferrous oxidation–xylenol orange assay, FOX-II [51,79,80,165]. The method consists of the oxidation (in acidic conditions at room temperature) of ferrous ions by hydroperoxides; the newly formed ferric acid can be bound with xylenol orange to produce a chromophore complex with strong absorbance between 540 and 600 nm [51,79,80,165,166]. The FOX-II assay is recognized as highly specific to hydroperoxides in general. Therefore, lipid isolation should be performed before analysis. Lipid hydroperoxide content is commonly expressed in cumene hydroperoxide (CHP) equivalents/g wet tissue [165]. Generally, the determination of LPO is carried out via a spectrophotometer at 530 nm [72,73], 535 nm [20,37,44,49,59,65,66,155,156,157,159,161,162], or 532 nm [13,19,38,41,47,48,50,55,74,75,76,77,154,155,158,160,167], or a fluorimeter at Ex/Em = 532/553 nm [17,63,153], 520/535 nm [81,168], or 530/560 nm [16,82,155,169]. The reaction was conducted in acidic media at 45 °C for 1 h [83], 90 °C for 45 min [49], 30 min [39], 1 h [47,50], 100 °C for 15 min [75,77,80], 20 min [37], 30 min [38,39], or 1 h [15,17,65,76].

Lipid peroxidation increased significantly with respect to the control in honeybees [63], tobacco worms [77], wax moths [72], and flies [20,44,45]. Hosamani and Muralidhara [48] reported a significant increase in fruit flies exposed to paraquat in the mitochondrial fraction, but a nonsignificant change in the cytosolic fraction. Exposure to heavy metals resulted in a significant increase in *A. mellifera* when exposed to Al [17], whereas LPO levels did not change significantly for similar exposures to Cd or Pb. In contrast, the exposure of *A. cerana*, as well as European corn borers [74] and fruit flies [47], to Cd significantly increased [56]. Exposure to TiO_2_ or Al_2_O_3_ nanoparticles did not affect the LPO in *O. fasciatus* [55].

#### 3.5.2. Protein Damage

An evaluation of the ROS-induced modifications of proteins in *A. thalassinus* [79,80], *A. mellifera* [10,17,57,62], *D. melanogaster* [47,49,51], *H. illucens* [53], *R. speratus* [38], and *S. exigua* [75] was performed in terms of carbonyl groups and thiol groups (Table A14). In particular, the formation of carbonyl compounds is the most general and widely used marker of severe protein oxidation [170,171,172]. The methods described in this review were based on reactions, under strongly acidic conditions, of carbonyl groups with 2,4-dinitrophenylhydrazine (DNPH) to form 2,4-dinitrophenylhydrazone (DNP), which has an absorbance at 366, 370, or 375 nm. The authors made minor modifications to this method; the following approaches differ in terms of the sample homogenization buffer utilized, such as PBS with or without additives (Triton X-100, CaCl_2_) [53,79] or Tris-HCl [10,38], as well as the solvent employed for rinsing the pellet-forming proteins from unreacted DNPH (ice cold acetone or ethanol/ethyl acetate, 1:1 mixture) [47,53,75]. The protein carbonyl content was quantified via an extinction coefficient of ε370 = 22 mM^−1^ cm^−1^ [51] or ε375 = 6.36 mM^−1^ cm^−1^ [75]. The total protein content is typically reported as the weight of total protein.

As described by Rovenko et al. [51], the assay of protein thiols was performed using Ellman’s method for determining sulfhydryl groups in tissues. The content was estimated by comparing total and low-molecular-weight thiols and analyzing the protein-containing and deproteinized fractions of the supernatants. The amount of protein in the supernatants was assayed after centrifugation with bovine serum albumin as the standard [173].

Research has revealed significant increases in the carbonyl group content in proteins compared with the control in fruit flies exposed to Cd(II) [47] or to a mixture of Fe(II) and rotenone, but not to Fe(II) or rotenone administered alone [49]. Honeybees exposed to heavy metals or metalloids presented increased protein damage when they were fed Cd(II) [17] or Se(IV)/(VI) [57], whereas Al(II) and Pb(II) did not affect proteins. Interestingly, a controlled sucrose/yeast diet, which produces a deficit of carbohydrates in D. melanogaster larvae, resulted in a decrease in protein carbonyls and protein thiols [51]. These findings provide valuable insights into the effects of ROS-induced protein modification in different organisms.

#### 3.5.3. DNA Damage

Studies on the effects of oxidative stress on insects, such as *A. domesticus* [78], *A. thalassinus* [79], *B. mori* [66], *C. riparius* [44], *G. mellonella* [72], *R. speratus* [38], *S. litura* [77], and *T. hispida* [16], involving DNA damage are shown in Table A15. The most commonly used approach is the comet assay, an alkaline gel electrophoresis-based method that can be used to measure DNA damage in individual eukaryotic cells [78,174,175,176,177]. This technique permits the evaluation of DNA strand break levels in samples with the assistance of image-analyzer software, examining the shape of single-cell nuclei unwound DNA spots forced to migrate. Parameters such as tail intensity, tail length, and tail moment (product of tail DNA% by tail length), percentage of severed cells (number of cells showing DNA damage/total number of cells), and olive moment (product of tail DNA% and distance of head and tail centroids) were employed in the statistical analysis; see Gyori et al. [178] for a more detailed description of the parameters.

The alkaline precipitation assay [179,180] was performed by Monteiro et al. [44]. Cell lysis was conducted via the addition of sodium dodecyl sulfate detergent (SDS) to NaOH, Tris, and EDTA. SDS-associated nucleoproteins and genomic DNA precipitate were obtained with the addition of KCl, and the separation of damaged DNA, which was collected from the supernatant, was achieved by centrifuging the mixture (8000× *g*, for 4 min). Hoechst dye was added to quantify strain breaks, and fluorescence emission was measured versus a whole mixture without a sample (a blank) at Ex/Em = 360/450 nm.

A different approach was used to determine the 8-hydroxy-2′-deoxyguanosine (8-OHdG) content of DNA. The concentration of 8-OHdG in the extracted insect DNA was determined via commercial kits (a competitive enzyme-linked immunosorbent assay utilizing a monoclonal antibody) [38,66,77].

An increase in DNA damage was observed in Lepidoptera exposed to agrochemicals [72,77] and to graphene oxide NPs [66], in house crickets exposed to diamond NPs [78], and in desert beetles exposed to textile industry soil (heavy metal contamination) [16]. A comparison of termites, queens, and workers exposed to UV-B revealed a significant increase in workers only [38]. A nonsignificant change in DNA strain breaks was observed in harlequin flies exposed to spinosad and indoxacarb [44].

## 4. Conclusions

Exposure to environmental pollutants can lead to numerous adverse effects, including oxidative stress phenomena. Using insects and particular biomarkers is useful for evaluating oxidative stress induced by environmental pollutants. The studies collected in this review indicate that the fundamental chemistry underlying the analytical methods has remained consistent since the last century. However, advancements in instrumentation and methodological protocols have enabled reductions in sample volumes and the use of safer reagents, aligning with modern green chemistry practices.

Nevertheless, sample preparation must be performed with caution to ensure sample stability and reduce the possibility of oxidative damage to tissues/cells/biomolecules during collection. Therefore, standardized sampling procedures and analytical methods for evaluating oxidative stress are urgently needed to determine the extent of this problem and fully understand its effects on all living organisms, including humans.

## Data Availability

No new data were created or analyzed in this study. Data sharing is not applicable to this article.

## Abbreviations

The following abbreviations are used in this manuscript:

8-OHdG	8-hydroxy-2′-deoxyguanosine
8-oxoG	8-oxoguanine
BaP	benzo[a]pyrene
CAT	catalase
CDNB	1-chloro-2,4-dinitrobenzene
CHP	cumene hydroperoxide
DCF-DA	2,7-dichlorofluorescein diacetate
DCHBS	3,5-dichloro-2-hydroxybenzenesulfonic acid
DHE	dihydroethidium
DNP	2,4-dinitrophenylhydrazone
DNPH	2,4-dinitrophenylhydrazine
DTNB	5,5′-dithiobis-(2-nitrobenzoic acid)
GONPs	graphene oxide nanoparticles
GPx	glutathione peroxidase
GPx-I	selenium-dependent glutathione peroxidase
GPx-II	selenium-independent glutathione peroxidase
GR	glutathione reductase
GSH	glutathione
GSSG	glutathione disulfide
GST	glutathione S-transferase
HRP	horseradish peroxidase enzyme
INT	p-iodonitrotetrazolium
LHP	lipid hydroperoxide
LPO	lipid peroxidation
MDA	malondialdehyde
MTLPs	metallothionein-like proteins
NBT	nitroblue tetrazolium
NBT+	formazan
OGG1	8-oxoguanine glycosylase
OPT	o-phthalaldehyde
PAH	polycyclic aromatic hydrocarbon
PC	protein carbonyls
PMSF	phenylmethylsulfonyl fluoride
PQ	paraquat
PT	protein thiols
RNS	reactive nitrogen species
ROS	reactive oxygen species
RS	reactive species
SOD	superoxide dismutase
SSA	sulfosalicylic acid
TBARS	thiobarbituric acid reactive substances
TCA	trichloroacetic acid
Tsh	total thiols
WST	tetrazolium salt
α-toc	alpha-tocopherol

## Appendix A

**Table A1:** Determination of nitrogen oxides (NOs) via the Griess colorimetric reaction test [88,89].

Insect	Sample Type	Exposure		Effect (Significance) ^a^	Method	Reading Wavelength	Reference
		Contaminant Type (Dose)	Period				
*Drosophila melanogaster* (fruit fly)	Whole body	Rotenone (50 μM)	7 days	Increased	150 μL 0.1 M potassium phosphate buffer (pH 7.4), 20 μL supernatant, and 25 μL Griess’ reagent were incubated at room temperature, in low or no illumination.	-	[15]
		*Syagrus coronata* fixed oil (0.2 mg/mL)		ns			
		Rotenone (50 μM) + *S. coronata* fixed oil (0.2 mg/mL)		ns			
*Drosophila melanogaster* (fruit fly)	Whole body	CdCl_2_ (0.05 mM)	7 days	Increased	Sample was incubated with Griess reagent in a 1:1 ratio at room temperature for 20 min.	550 nm	[47]
		hesperidin (50 and 100 μM)		ns			
		CdCl_2_ (0.05 mM) + hesperidin (50 and 100 μM)		ns			
*Drosophila melanogaster* (fruit fly)	Head	FeSO_4_ (1.0 and 10.0 μM)	10 days	ns	Sample was incubated with Griess reagent in a 1:1 ratio at room temperature for 20 min.	550 nm	[49]
		Rotenone (50.0 μM)		ns			
		FeSO_4_ (1.0 and 10.0 μM) + rotenone		Increased			
*Locusta migratoria*	Testicular tissues	Al_2_O_3_ NPs (0.03 mg/g body weight)	Single dose injection	Increased (*)	Nitric oxide assay kit (ab65328, Abcam Co., Berlin, Germany).	-	[13]
		Al_2_O_3_ NPs + *Periplaneta americana* extract (0.05 mg/g body weight)		ns			

^a^ Analysis of variance (ANOVA) was performed to highlight statistical differences between treatment and control groups at a confidence level of 95% (*p* < 0.05), except when differently noted: *, *p* < 0.001; ns, no significant variation.

**Table A2:** Determination of reactive oxygen species (ROS).

Insect	Sample Type	Exposure		Effect (Significance) ^a^	Method	Reading Wavelength	Reference
		Contaminant Type (Dose)	Period				
*Bombyx mori* (silkworm), larvae	Midgut, Malpighian tubules, and fat body with adhered tissues	Polystyrene nanoplastics (0.25 mg/0.5 g diet)	10 days for behavior analysis and 21 days for biochemical analyses	ns	Sample in 100 μL of PBS and 8.3 μL of DCFH-DA (10 mg mL^−1^ in DMSO) were incubated for 30 min at 37 °C.	Ex/Em = 485/530 nm	[65]
*Bombyx mori* (silkworm), cell culture and female adults	Ovary cell line	Graphene oxide nanoparticles (GONPs 25 mg/L)	24 h, 48 h and 72 h	Increased (*)	DCFH-DA kit (Nanjing Jiancheng Bioengineering Institute, Nanjing, China)	Ex/Em = 500/525 nm	[66]
	Ovarian tissues of female adults	GONPs (25 mg/L)	48 h	Increased (*)			
*Drosophila melanogaster* (fruit fly), cell culture	-	Sb (1.2 mg/mL)	48 h	Increased (**)	DCFH-DA assay kit (S0033, Beyotime Biotechnology, Shanghai, China). Cells were incubated with DCFH-DA probe for 30 min in the dark. After incubation, the cells were washed in PBS.	Ex/Em = 488/525 nm	[21]
		Sb (1.2 mg/mL) + N-acetylcysteine (NAC at 0.15, 0.75 and 1.50 mg/mL)		ns			
		Sb (1.2 mg/mL) + glutathione (GSH at 0.15, 0.75 and 1.50 mg/mL)		ns			
*Drosophila melanogaster* (fruit fly)	Whole body (Cytosolic and mitochondrial fractions)	Paraquat (PQ, 10, 20, and 40 mM in 5% sucrose solution)	24 h	Increased with PQ at 20 and 40 mM	2 mL Locke’s buffer (pH 7.4), 0.1 mL cytosol (100 μg protein), and 10 μL DCFH-DA (5μM) were incubated for 15 min at room temperature.	Ex/Em = 484/530 nm	[48]
*Drosophila melanogaster* (fruit fly)	Whole body	*Eugenia uniflora* leaves essential oil (3, 15 and 30 μg/mL)	3, 6 and 12 h	Increased (3 h)	100 μL sample were incubated in the presence of 5 μM DCF-DA at 37 °C for 1 h [92].	Ex/Em = 485/530 nm	[50]
*Spodoptera exigua* (beet armyworm) cell culture	Cells	Camptothecin (CPT) and hydroxyl-camptothecin (HCPT) at 0.1, 1, 5 and 10 μM	2, 4, 6, 12, 24, and 48 h	Increased	500 μL PBS containing 10 μM DCFH-DA were added to the collected cells and then incubated at 37 °C for 20 min in the dark. After incubation, the cells were washed in PBS.	Ex/Em = 485/528 nm	[75]
*Tenebrio molitor* (mealworm larvae)	Whole body	Polyethylene (3 g)	24 days	Increased	DHE assay kit (Biolab Technology Co., Ltd., Beijing, China). Supernatant was incubated at 37 °C for 30 min.	Ex/Em = 488–535/610 nm	[41]
		Polystyrene (3 g)		Increased			
		polyvinyl chloride (3 g)		Increased			

^a^ Analysis of variance (ANOVA) was performed to highlight statistical differences between treatment and control groups at a confidence level of 95% (*p* < 0.05); *, *p* < 0.01; **, *p* < 0.001; ns, no significant variation.

**Table A3:** Determination of hydrogen peroxide.

Insect	Sample Type	Exposure		Effect (Significance ^a^)	Method	Reading Wavelength	Reference
		Contaminant Type (Dose)	Period				
*Apis mellifera* (honeybee)	Whole body	Environmental pollutants produced by waste burning (metals)	1 week	ns in control bees before and after the fire	Colorimetric/Fluorometric assay kit (ab102500, BioVision Abcam, Cambridge, United Kingdom).	570 nm	[10]
*Apis mellifera* (honeybee)	Whole body	Se(IV) (0.6, 6, 60, 600 μg/mL)	2 days	Increased (*, 60 μg/mL; 600 μg/mL)	Colorimetric/Fluorometric assay kit ab102500 (BioVision Abcam)	570 nm Ex/Em = 535/587 nm	[57]
		Se(VI) (0.6, 6, 60, 600 μg/mL)		ns			
*Bombyx mori* (silkworm), cells culture and female adults	Ovarian tissues of female adults	Graphene oxide nanoparticles (GONPs, 25 mg/L)	48 h	Increased	Assay kit (Beyotime Biotechnology)	405 nm	[66]
*Drosophila melanogaster* (fruit fly)	Whole body	CdCl_2_ (0.05 mM)	7 days	Increased	Wolff’s method [94]: 590 μL FOX 1 + 10 μL sample and 30 min of incubation at room temperature.	560 nm	[47]
		hesperidin (50 and 100 μM)		ns			
		CdCl_2_ (0.05 mM) + hesperidin (50 and 100 μM)		ns			
*Drosophila melanogaster* (fruit fly)	Whole body (cytosolic fraction)	Paraquat (PQ, 10, 20, and 40 mM in 5% sucrose solution)	24 h	Increased with PQ at 20 and 40 mM	Wolff’s method [94]: 100 μg cytosolic protein was added to 1 mL of FOX reagent and incubated for 30 min at room temperature.	560 nm	[48]
*Drosophila melanogaster* (fruit fly)	Head	FeSO_4_ (1.0 and 10.0 μM)	10 days	ns	Wolff’s method [94]: 290 µL FOX 1 + 10 µL sample and incubated for 30 min at room temperature.	560 nm	[49]
		Rotenone (50.0 μM)		ns			
		FeSO_4_ (1.0 and 10.0 μM) + rotenone		Increased			
*Hermetia illucens* (black soldier fly), larvae	Gut tissues	Contaminated soils with 2% solution of Cd, Fe or Pb, or catechol, or organic compound used for pesticide production mixed with organic wastes (fruit, vegetable or kitchen) in a 2:0.5 ratio	7 days	All values depict significant differences among control and the different waste types except fruit wastes + Cd, kitchen wastes or kitchen wastes + Cd	Potassium iodide assay [95]: 0.5 mg of sample + 1 mL of potassium phosphate buffer (10 mM; pH = 7.0) containing 0.25 mL Trichloroacetic acid (0.1%, *w*/*v*), 0.5 mL KI (1 M)	240 nm	[53]

^a^ Analysis of variance (ANOVA) was performed to highlight statistical differences between treatment and control groups at a confidence level of 95% (*p* < 0.05); *, *p* < 0.01; ns, no significant variation.

**Table A4:** Determination of superoxide.

Insect	Sample Type	Exposure		Effect (Significance ^a^)	Method	Reading Wavelength	Reference
		Contaminant Type (Dose)	Period				
*Aedes aegypti* “Bora-Bora” (mosquito), larvae	Whole body	0.5, 5 and 50 µg/L of fluoranthene or benzo[a]pyrene (BaP) or to a 1:1 wt:wt mix of both 0.5, 5 and 50 µg/L of fluoranthene or benzo[a]pyrene (BaP) or to a 1:1 wt:wt mix of both (0.25, 2.5 and 25 µg/L of each pollutant for a final concentration of 0.5, 5 and 50 µg/L of total PAHs, respectively)	24 h (1 h irradiation)	Higher PAH concentrations reduced ROS production, particularly for BaP and the PAH mixture, regardless of UV exposure. In PAH-unexposed larvae, UV exposure caused an 80% increase in ROS ^b^.	Chemical reduction of the redox dye NBT [97]: 20 larvae were added to 1 mL of a 1 mg/L NBT solution for 2 h in the dark at room temperature. Larvae were washed twice in ethanol, air-dried, and the formazan produced was then dissolved in 50 μL KOH (2 M) and 75 μL DMSO.	630 nm	[42]
*Drosophila melanogaster* (fruit fly)	Whole body (Mitochondrial fraction)	Paraquat (PQ, 10, 20, and 40 mM in 5% sucrose solution)	24 h	Increased with PQ at 20 and 40 mM	DHE oxidation method [96], 25 μg of mitochondrial protein.	Ex/Em = 490/590 nm	[48]

^a^ Analysis of variance (ANOVA) was performed to highlight statistical differences between treatment and control groups at a confidence level of 95% (*p* < 0.05), except when differently noted: ^b^, generalized linear models (GLMs).

**Table A5:** Determination of catalase (CAT) activity.

Insect	Sample Type	Exposure		Effect (Significance) ^a^	Method	Reading Wavelength	Reference
		Contaminant Type (Dose)	Period				
*Acheta domesticus* (house cricket)	Hemolymph	Nanodiamonds (NDs, 20 and 200 µg/g food)	7 weeks	ns	H_2_O_2_ decomposition [99]: supernatant + 0.05 M phosphate Sørensen buffer (pH 7.4) were incubated at 37 °C for 10 min. Then, 10 mM of H_2_O_2_ were added.	40 s (with an 8 s interval) at 240 nm	[78]
	Head			ND 200 increased (*)			
	Gut			ND 200 increased (*)			
	Gonads			ND 200 increased (*)			
	Fat body			ns			
*Acridoidea* (grasshoppers)	Gut	Azadirachtin (1, 5, 10, 15, and 20 ppm)	One application per 25 g of food plants	Increased (**)	Gel zymography assay [100]: The nondenaturing (8%) acrylamide gel + 25 µg proteins in distilled water for 10 min. The gel was shaken in 100 mL distilled water + 100 µL H_2_O_2_ for 10 min and then it was washed with distilled water for 5 min.	-	[84]
*Aiolopus thalassinus* (grasshopper), nymph	Brain	Heavy metals (Cu, Zn, Pb, Cd), PO_4_^3−^, and SO_4_^2−^ in soil samples from low and high polluted sites	-	The level of CAT activity from the high polluted site revealed a significant decrease (*) compared to individuals from the control and low-pollution sites. The highest value of CAT was found in the gut of nymphs from the low polluted site	H_2_O_2_ decomposition [99]	240 nm	[79]
	Thoracic muscles						
	Gut						
*Aiolopus thalassinus* (grasshopper)	Brain, thoracic muscles, and gut	Heavy metals (Cu, Zn, Pb, Cd), PO_4_^3−^, and SO_4_^2−^ in soil samples from low and high polluted sites	-	The level of CAT activity revealed a significant (*) decreasing effect compared to the individuals from the control site. A strong inhibition of CAT activity was especially observed in the case of females.	H_2_O_2_ decomposition [99]: 510 µL supernatant + 3060 µL potassium phosphate buffer (50 mM, pH 7.0) + 40 µL H_2_O_2_ (10 mM).	240 nm	[80]
*Anaceana globulus* (water scavenger beetle	Whole body	Heavy metal polluted water (Cu, Zn, Fe, Mn, Pb, Co and Cd)		Highly significant in samples collected from the polluted site in compare the reference one.	H_2_O_2_ decomposition [99]	240 nm	[39]
*Apis mellifera* (honeybee)	Thorax–abdomen (cytosolic fraction)	Sited with different degrees of environmental pollution mainly related to agricultural and industrial activities		Only the CAT enzyme showed slightly higher activity in bees from urban areas compared to those collected from natural areas	H_2_O_2_ decomposition: the assay was carried out in phosphate buffer (pH 7) and H_2_O_2_ 24 mM.	240 nm	[11]
*Apis cerana*	Whole body	CdCl_2_ ((12.5, 25, 50, 100, 200, 400, and 800 mg/L)	48 h	ns	CAT-assay kit (Nanjing Jiancheng Bioengineering Institute)	405 nm	[56]
*Apis mellifera* (honeybee)	Whole body	Pb(II) (15 μg)	One dose of 8 μL	ns	H_2_O_2_ decomposition [106]: 5 µL sample + 600 μL reaction mixture (150 μL of 3% *v*/*v* H_2_O_2_ in 400 μL of phosphate buffer solution pH = 7) + incubated for 10 min at 22 °C.	240 nm	[58]
*Apis mellifera* (honeybee), queens and foragers	Hemolymph	Imidacloprid (5 and 200 ppb)	-	Significant differences in function of age, role, and concentration (*)	H_2_O_2_ decomposition [99,105]	240 nm	[59]
*Apis mellifera* (honeybee)	Head	Thiamethoxam Flusilazole (FSZ) +combination (LC50 values)	2-day and 4-day intervals	The CAT activity was steeply diminished under the high dose of FSZ exposure than at the baseline level.	Commercial kit	-	[60]
*Bombyx mori* (silkworm) larvae	Midgut, Malpighian tubules, and fat body with adhered tissues	Polystyrene NPs (0.25 mg/0.5 g diet)	10 days	ns	Decomposition of H_2_O_2_ (50 mM) in 100 mM potassium phosphate buffer (pH 7) [98,109].	240 nm	[65]
*Bombyx mori* (silkworm), female adults	Ovarian tissues of female adults	Graphene oxide NPs (GONPs, 25 mg/L)	48 h	Increased	CAT-assay kit (Nanjing Jiancheng Bioengineering Institute)	-	[66]
*Bombyx mori* (silkworm), larvae	Hemolymph	ZnO-NPs (50 and 100 μg/mL)	The larvae were fed three times per day.	Increased	Phosphate buffer + H_2_O_2_ were added to hemolysate [73].	3 min at 240 nm	[67]
*Chironomida*, *Gomphus*, and *Lestes*	Whole body	The study was conducted mainly on three rivers in polluted area	-	The highest CAT activities were recorded in the turbid river, while the lowest ones were recorded in the clear river.	CAT-assay kit (ECAT-100, Bio Assay Systems, Hayward, CA, USA): 10 µL supernatant + 10 µL assay buffer + 1 µL 4.8 mM H_2_O_2_ (by mixing 5 µL 3% H_2_O_2_ and 914 µL dH_2_O) incubate for 30 min at room temperature.	470 nm	[83]
*Chironomus riparius* (harlequin fly), larvae	Whole body	Esfenvalerate (ESF, 0.075, 0.3 and 1.2 mg/L)	24 h	Decreased at 0.3 and 1.2 mg/L ESF	H_2_O_2_ decomposition [101].	240 nm	[20]
*Chironomus riparius* (harlequin fly), larvae	Whole body	Spinosad (1, 2, 4, 8, 16, 32, 64, 128, and 256 μg/L)	48 h	ns	H_2_O_2_ decomposition [101] 10 μL of protein, 190 μL K_2_HPO_4_ (pH = 7) and 0.5 μL H_2_O_2_. The blank was done using 10 μL of homogenization buffer instead of protein.	1 min at 240 nm	[44]
		Indoxacarb (0.5, 1, 2, 4, 8, 16, 32, 64, and 128 μg/L)		ns			
*Chironomus riparius* (harlequin fly), larvae	Whole body	Tetracycline (2, 20, and 200 μg/L)	48 h	The significantly highest value is for TC at 1.83 ug/L	H_2_O_2_ decomposition [101].	240 nm	[45]
*Drosophila melanogaster* (fruit fly)	Head	Brilliant black PN (azo dye, E151; 1, 2.5, and 5 mg/mL)	5 mL	Decreased at 5 mg/mL	H_2_O_2_ decomposition [99].	240 nm	[19]
*Drosophila melanogaster* (fruit fly)	Whole body	CdCl_2_ (0.05 mM)	7 days	Decreased	H_2_O_2_ decomposition [99]: 50 mM potassium phosphate buffer (pH 7.0), 300 mM H_2_O_2_ and sample (1:50 dilution).	2 min at 240 nm	[47]
		hesperidin (50 and 100 μM)		ns			
		CdCl_2_ (0.05 mM) + hesperidin (50 and 100 μM)		Decreased with hesperidin 50 μM			
*Drosophila melanogaster* (fruit fly)	Whole body (Cytosolic fraction)	Paraquat (PQ, 10, 20, and 40 mM in 5% sucrose solution)	24 h	Increased at PQ 20, and 40 mM	H_2_O_2_ decomposition [99].	3 min at 240 nm	[48]
*Drosophila melanogaster* (fruit fly)	Head	FeSO_4_ (1.0 and 10.0 μM)	10 days	ns	H_2_O_2_ decomposition [99]: 10 µL of sample (1:50 dilution) and 50 mM potassium phosphate buffer (pH 7.0) followed with 300 mM H_2_O_2_.	2 min at 240 nm	[49]
		Rotenone (50.0 μM)		ns			
		FeSO_4_ (1.0 and 10.0 μM) + rotenone		Decreased			
*Drosophila melanogaster* (fruit fly)	Whole body	*Eugenia uniflora* leaves essential oil (3, 15 and 30 μg/mL)	3 and 12 h	ns	H_2_O_2_ decomposition [99]: 50 mM phosphate buffer pH 7.0, 0.5 mM EDTA, 10 mM H_2_O_2_, 0.012% Triton X100.	240 nm	[50]
*Drosophila melanogaster* (fruit fly)	Whole body	Experimental food: 4% yeast, 0.25%, 1%, 4%, 10% or 20% sucrose, 1.25% (*w*/*v*) agar and 0.4% (*v*/*v*) propionic acid (carbohydrate deficit)	25 mL	Catalase activity had the lowest value in male flies grown on 0.25% sucrose and was not affected by diets used in females	-	-	[51]
*Galleria mellonella* (wax moth), larvae	Midgut	CuO-NPs (size < 50 µm, 10 μg/L)	15 days	Increased	H_2_O_2_ decomposition [103].	240 nm	[68]
	Fat body			Increased			
*Galleria mellonella* (wax moth), larvae	Midgut Fat body	Al_2_O_3_ nanopowder (size: <50 nm) and micron powder (size: 45–75 μm) at 10, 50, and 100 μg/mL	One injection of 10 μL of suspension	Increased at 50 and 100 μg/mL Al_2_O_3_ micropowder; decreased (*) in Al_2_O_3_ NPs (in fat body decreased at 50 μg/mL and increased at 100 μg/mL)	H_2_O_2_ decomposition [103].	240 nm	[69]
*Galleria mellonella* (wax moth), larvae	Midgut or fat body	CuSO_4_ (10 mg/L)	72 h	Increased (midgut)	H_2_O_2_ decomposition [103].	240 nm	[70]
		ZnCl_2_ (30 mg/L)		Decreased			
		CuSO_4_ + ZnCl_2_ (1:1 ratio)		Decreased			
*Galleria mellonella* (wax moth), larvae	Tissue	Juglone at LC10 (0.5 mg), LC30 (1.5 mg) and LC50 (2.3 mg) in a 2 g diet.	-	Increased at LC30 and LC50, while decreased (**) at LC10	H_2_O_2_ decomposition [100].	3 min at 240 nm	[72]
*Galleria mellonella* (wax moth), larvae	Hemolymph	Azadirachtin (0.5, 1, 1.5, 2, and 3 μg/larva)	24 and 72 h post treatment	Decreased significantly at 1, 1.5, and 2 μg/larva doses after 24 h, but increased at all doses except for 0.5 μg/larva at 72 h post treatments. The effect of AZA on CAT activity in larval hemolymph was dose- and time-dependent.	H_2_O_2_ decomposition [100].	3 min at 240 nm	[73]
*Leptinotarsa decemlineata* (Colorado potato beetle), larvae and adults	Whole body	Glyphosate based herbicide (450 g/L)	-	CAT activity increased with body mass in the adult beetles in GBH treatment	CAT-assay kit (CAT100, Sigma-Aldrich, St. Louis, MO, USA) with modifications [111,112]: 0.6 mg/mL sample + assay buffer (50 mM KF + 0.1% TritonX100, pH 7.0), chromogen reagent (0.25 mM 4-aminoantipyrene + 2 mM 3,5-dicloro-2-hydroxybenzenesulfonic acid in 150 mM potassium phosphate buffer, pH 7.0), peroxidase solutions (from horseradish), stop solution (15 mM NaN_3_), and 200 mM and 10 mM H_2_O_2_.	520 nm	[40]
*Locusta migratoria* (Locust)	Testicular tissues	Al_2_O_3_ NPs (0.03 mg/g body weight)	Single dose injection	Increased (**)	H_2_O_2_ decomposition [99].	240 nm	[13]
		Al_2_O_3_ NPs + *Periplaneta americana* extract (0.05 mg/g body weight)		ns			
*Oncopeltus fasciatus* (large milkweed bug)	Whole body	FeSO_4_·7H_2_O + ascorbic acid (2000 and 5000 mg/L)	96 h	Increased	H_2_O_2_ decomposition [99].	1 min at 240 nm	[54]
*Periplaneta americana* (American cockroaches)	Whole body	Bendiocarb (0.1 nM)	1 mL	Increased (**)	H_2_O_2_ decomposition [107]: the diluted sample was added to phosphate buffer and 30 mM H_2_O_2_.	3 min, at 1 min intervals, at 240 nm	[37]
*Reticulitermes speratus* (termite: workers, queens, soldiers, and nymphs)	Whole body	UV-B (312 nm, 10.4 kJ/m^2^)	20 min	Increased in queens	15 μg total protein + reaction buffer containing 50 mM Tris-HCl (pH 7.5), 2.5 mM EDTA and 10 mM H_2_O_2_ [110].	240 nm	[38]
*Stenopsyche marmorata* (caddisfly) larvae	Whole body (except head, tail, and gut)	Anthropogenic pollutants by effluents of wastewater treatment plants (WWT)	-	WWTP effluent induced CAT	H_2_O_2_ decomposition [102].	240 nm	[82]
*Spodoptera litura* (tobacco worm) larvae	Gut	Fipronil (20, 40, 60 and 80 mg/L)	24–72 h	At the lower concentration and early exposure, time, CAT activity did not increase significantly. As the exposure time increased, the CAT activity increased significantly during the 24 h and 48 h and then decreased compared to the control after 72 h of exposure.	H_2_O_2_ decomposition [104].	-	[77]
*Trachyderma hispida* (desert beetle)	Midgut	The polluted site is characterized by the presence of agricultural land and high concentrations of elements (especially P, S, and Na) due to the proximity of a textile plant.	-	Decreased in polluted site	H_2_O_2_ decomposition [99].	240 nm	[16]

^a^ Analysis of variance (ANOVA) was performed to highlight statistical differences between treatment and control groups at a confidence level of 95% (*p* < 0.05), except when differently noted: *, *p* < 0.01; **, *p* < 0.001; ns, no significant variation.

**Table A6:** Determination of superoxide dismutase (SOD) activity.

Insect	Sample Type	Exposure		Effect (Significance) ^a^	Method	Reading Wavelength	Reference
		Contaminant Type (Dose)	Period				
*Acridoidea* (Grasshopper)	Gut	Azadirachtin (1, 5, 10, 15, and 20 ppm)	One application per 25 g of food plants	Increased (**) at 10 mg/kg	Gel zymography [124]: supernatant from tissue homogenate was electrophoresed in riboflavin gel at 4 °C. Gels were first incubated in 2.4 mM NBT in deionized water for 15 min and then in 0.028 mM riboflavin/280 mM N,N,N,N-tetramethyl-ethylenediamine (TEMED) in 50 mM potassium phosphate buffer (pH 7.8) for 15 min in the darkroom. After washing, the gel was illuminated under fluorescent light to get a distinct SOD activity band.	-	[84]
*Aiolopus thalassinus*, nymph	Brain	Environmental pollution (low or high level)	-	Increased ^b^ (low level)	Epinephrine autoxidation [116].	480 nm	[79]
	Thoracic muscles			ns			
	Gut			ns			
*Aiolopus thalassinus*	Brain, thoracic muscles, and gut	Environmental pollution: fertilizer industry surroundings	-	Sex-, tissue-, and distance-dependent	Epinephrine autoxidation [116]: 402 µL of a sodium carbonate buffer (200 mM; pH 10.0), 35 µL of EDTA (10 mM), 87 µL of the supernatant of the appropriate tissue and 2835 µL of freshly prepared epinephrine (15 mM).	480 nm	[80]
*Apis cerana* (honeybee)	Whole body	CdCl_2_ (5.1 mg/L)	14 days	Decreased	SOD assay kit (A001, Nanjing Jiancheng Bioengineering Institute)	550 nm	[56]
*Apis mellifera* (honeybee)	Thorax–abdomen (cytosolic fraction)	Environmental pollution	-	-	50 mM Na_2_CO_3_ buffer pH 10 with 0.1 mM EDTA, 500 mM cytochrome C, 1 mM hypoxanthine, and xanthine oxidase.	550 nm	[11]
*Apis mellifera* (honeybee), queens and foragers	Hemolymph	Imidacloprid (5 and 200 µg/kg)	1 month	Decreased (5 µg/kg *)	p-iodonitrotetrazolium/ xanthine/xanthine oxidase method [121].	505 nm	[59]
*Apis mellifera* (honeybee)	Head	Flusilazole (2.3–37 mg/L)	7 days	Decreased (37 mg/L)	SOD assay kit (A001, Nanjing Jiancheng Bioengineering Institute).	-	[60]
		thiamethoxam (0.3–5.5 μg/L)		Decreased (1.3 μg/L)			
		flusilazole (0.06–0.92 mg/L) + thiamethoxam (0.009–0.014 μg/L)		Modulated			
*Atta sexdens* (leafcutter ant), workers	Fat body	Sulfaramid (0.005% in acetone)	24–48 h	ns	Pyrogallol autoxidation inhibition in EDTA (pH 8.2) [115]: 20 μL of supernatant from samples and 180 μL 0.2 mM pyrogallol.	20 min at 420 nm	[64]
		Fipronil (0.001% in acetone)	24–72 h	Increased (24 h)			
		Mg-1 complex (2 g/L)	48–96 h	Decreased (96 h)			
*Bombyx mori* (silkworm) larvae	Midgut, Malpighian tubules, and fat body with adhered tissues	Polystyrene nanoplastics (0.5 mg/kg in 0.5 g of feeding)	21 days	Decreased	The activity was quantified measuring the degree of inhibition of cytochrome C (0.3 mM) in 50 mM phosphate buffer + EDTA (0.1 mM), generated by the reaction mix of 1.5 mM hypoxanthine and xanthine oxidase 56.1 mU mL^−1^.	550 nm	[65]
*Bombyx mori* (silkworm), female adults	Ovarian tissues	Graphene oxide nanoparticles (GONP) (25 mg/L in 100 µL of culture medium)	24 h	Increased	SOD assay kit (Beyotime Biotechnology).	-	[66]
*Chironomus riparius* (harlequin fly), larvae	Whole body	Spinosad (0.5–8 μg/L)	48 h	ns	Cytochrome c reduction [122].	5 min at 550 nm	[44]
		Indoxacarb (2–8 μg/L)		ns			
*Chironomus riparius* (harlequin fly), larvae	Whole body	Tetracycline (1.83–174 μg/L)	48 h	Decreased (18.5 µg/L)	Inhibition of cytochrome c reduction in the presence of the hypoxanthine/xanthine oxidase O_2_−radical dot generator system [122].	-	[45]
*Chironomidae*, *Gomphus*, and *Lestes*	Whole body	Comparison of different rivers on different insect genera	-	-	20 μL of standard + 20 µL sample + 160 µL of assay buffer, 5 µL of xanthine + 5 µL of WST-1 + 20 µL xanthine oxidase enzyme.	440 nm	[83]
*Drosophila melanogaster* (fruit fly)	Head	Brilliant black PN (azo dye, E151) (1–5 mg/L)	up to development of the larvae	Increased (5 mg/L)	Nitroblue tetrazolium reduction rate [120].	560 nm	[19]
*Drosophila melanogaster* (fruit fly), cells	-	Sb(III) (Potassium antimonyl tartrate trihydrate) (1.2 mg/mL)	entire growth cycle, from embryos to hatching adults	Increased **	Epinephrine autoxidation [116].	4 min at 30 °C at 480 nm	[21]
*Drosophila melanogaster* (fruit fly)	Whole body (cytosolic or mitochondrial fraction)	Paraquat (PQ) (10–40 mM)	24 h	Increased (20 mM)	Inhibition of quercetin autooxidation [117]: 1 mL reaction contained 3–5 μg protein; 0.016 M sodium phosphate buffer (pH 7.8); 8 mM TEMED, 0.08 mM EDTA, and the reaction was started by adding 0.15% quercetin dissolved in dimethyl formamide.	3 min at 406 nm	[48]
*Drosophila melanogaster* (fruit fly)	Whole body	*Eugenia uniflora* leaves essential oil (3 mg/L)	3, 6, 12 h	Increased (12 h)	Inhibition of quercetin autooxidation [117].	406 nm	[50]
*Drosophila melanogaster* (fruit fly)	Whole body	Sucrose/yeast larval diet (0.25–20% as sucrose)	up to pupation (96 h)	-	Inhibition of quercetin autooxidation [118].	-	[51]
*Galleria mellonella* (wax moth), larvae	Midgut	CuO-NPs (size <50 µm) (10 µg/L)	15 days	ns	Decrease in cytochrome c by the xanthine oxidase/hypoxanthine system [122].	-	[68]
	Fat body			Decreased			
*Galleria mellonella* (wax moth), larvae	Midgut Fat body	Al_2_O_3_-NPs (<50 µm, or <50 nm) (10–100 mg/L in 10 µL volume)	Single injection	NPs size-, dose-, and tissue-dependent	Decrease in cytochrome c by the xanthine oxidase/hypoxanthine system [122].	-	[69]
*Galleria mellonella* (wax moth)	Midgut	CuSO_4_ (10 mg/L)	72 h	Increased	Decrease in cytochrome c by the xanthine oxidase/hypoxanthine system [122].	550 nm	[70]
		ZnCl_2_ (30 mg/L)		Increased			
		CuSO_4_ (10 mg/L) + ZnCl_2_ (30 mg/L)		Decreased			
	Fat body	CuSO_4_ (10 mg/L)		Decreased			
		ZnCl_2_ (30 mg/L)		Decreased			
		CuSO_4_ (10 mg/L) + ZnCl_2_ (30 mg/L)		Decreased			
*Galleria mellonella* (wax moth)	Hemolymph	Gibberellic acid (GA_3_) (0.05–5 g/kg)	up to a weight target of approx. 18 g	Modulated	SOD assay kit (Cayman Chemical, Ann Arbor, MI, USA)—xanthine/xanthine oxidase systems.	450 nm	[71]
*Galleria mellonella* (wax moth), larvae	Whole body	Juglone (0.25–1.15 mg/g)	up to a weight target of approx. 18 g	Modulated	SOD assay kit (Cayman Chemical)—xanthine/xanthine oxidase systems.	450 nm	[72]
*Galleria mellonella* (wax moth), larvae	Hemolymph	Azadirachtin (0.5–3 µg/larva)	24 h delayed analysis	Decreased (1 µg/L)	SOD assay kit (Cayman Chemical) using xanthine and xanthine oxidase systems.	450 nm	[73]
			72 h delayed analysis	Decreased (0.5 µg/L)			
*Hermetia illucens* (black soldier fly), larvae	Gut tissues	Vegetable, fruit, or kitchen wastes + Cd, Fe, Pb, or Catechol	-	-	Epinephrine autoxidation [116]: 1.5 mL Na_2_CO_3_ buffer (200 mM; pH 10.0), 0.5 mL EDTA (10 mM), 0.5 mL of the supernatant, and 0.5 mL freshly prepared epinephrine (15 mM).	480 nm	[53]
*Hydropsyche pellucidula* (net-spinning caddisfly), larvae	Whole body	Polypropylene microplastics (125 µg/L)	10 days	ns ^b^	Tris-EDTA buffer (pH 8.2) and pyrogallol [114].	420 nm	[81]
*Leptinotarsa decemlineata* (Colorado potato beetle), larvae and adults	Whole body	Glyphosate based herbicide (long-term contaminated-soil growth potatoes)	9 days	Increased ^c^	SOD assay kit (19160, Sigma-Aldrich).	450 nm	[40]
*Locusta migratoria* (Locust)	Testicular tissues	Al_2_O_3_ NPs (0.03 mg/g body weight)	single injection	Increased **	SOD assay kit (MBS433565, MyBioSource Co., San Jose, CA, USA).	560 nm	[13]
		Al_2_O_3_ NPs (0.03 mg/g body weight) + PAE (*Periplaneta americana* extract) (0.05 mg/g body weight)		ns			
*Stenopsyche marmorata* (caddisfly), larvae	Whole body (except head, tail, and gut)	Environmental pollution: wastewater treatment plants (comparison of spots and seasons)	-	-	SOD assay kit-WST (Dojindo Molecular Technologies, Inc., Kumamoto, Japan).	450 nm	[82]
*Spodoptera litura* (tobacco worm) larvae	Gut	Fipronil (FP) (20–80 mg/L)	12–72 h	Modulated	Pyrogallol method [115]: 2.8 mL of reaction mixture contains Tris-EDTA buffer (pH 8.2), and 50 µL of the supernatant. The final volume was raised to 2.9 mL with Tris-EDTA buffer, and 100 µL of pyrogallol was added to start the reaction.	3 min at 440 nm	[77]
*Tenebrio molitor* (mealworm larvae)	Whole body	polyethylene (3 g per 100 larvae)	24 h (dose refreshed every 4 days)	Increased	SOD assay kit (Abbkine Scientific Co., Ltd., Shanghai, China).	450 nm	[41]
		polystyrene (3 g per 100 larvae		Increased			
		polyvinyl chloride (3 g per 100 larvae)		Increased			
*Trachyderma hispida* (desert beetle)	Midgut	Textile industry soil (metal contaminants)	-	Decreased ^d^	Autoxidation of 5,6,6a,11b—tetrahydro-3,9,10-trihydroxybenzo[*c*]fluorene (BXT) [126]: pH 8.8, 37 °C, in 50 mM air-saturated 2-amino-2-methyl-1,3-propanediol buffer containing 3 mM boric acid and 0.1 mM diethylenetriaminepentaacetic acid.	1 min at 525 nm	[16]

^a^ Analysis of variance (ANOVA) was performed to highlight statistical differences between treatment and control groups at a confidence level of 95% (*p* < 0.05), except when differently noted: ^b^, Student’s *t*-test; ^c^, Kruskal–Wallis test; ^d^, Gaussian distribution and identity link function (GLMM); *, *p* < 0.01; **, *p* < 0.001; ns, no significant variation.

**Table A7:** Determination of glutathione s-transferase (GST) activity.

Insect	Sample Type	Exposure		Effect (Significance) ^a^	Method	Reading Wavelength	Reference
		Contaminant Type (Dose)	Period				
*Aedes aegypti* (mosquito), larvae	Whole body	*A*: Fluoranthene (0.5–50 μg/L)	1 h irradiation	Modulated	GSH-CDNB assay [86]: the reaction mixture contained 20 μg protein from the cytosolic fraction, 1.3 mM reduced glutathione and 1 mM CDNB in 0.1 M phosphate buffer (pH 6.9).	1 min at 340 nm	[42]
		*B*: benzo[a]pyrene (0.5–50 μg/L)		ns			
		*C*: Fluoranthene (0.25–25 μg/L) + benzo[a]pyrene (0.25–25 μg/L)		ns			
		*A* + UV-A (375 nm, 30 W/m^2^)		Modulated			
		*B* + UV-A (375 nm, 30 W/m^2^)		ns			
		*C* + UV-A (375 nm, 30 W/m^2^)		ns			
*Apis cerana* (honeybee)	Whole body	CdCl_2_ (5.1 mg/L)	14 days	Increased	GST assay kit (Nanjing Jiancheng Bioengineering Institute).	412 nm	[56]
*Apis mellifera* (honeybee)	Thorax–abdomen (cytosolic fraction)	Environmental pollution	-	-	NaH_2_PO_4_ + Na_2_HPO_4_ 100 mM buffer (pH 6.5) with GSH and CDNB 2 mM [126]	340 nm	[11]
*Apis mellifera* (honeybee) queens and foragers	Hemolymph	Imidacloprid (5 and 200 μg/kg)	1 month	Age-, role-, and concentration-dependent	GSH-CDNB assay [105]: 215 μL of 0.1 M sodium phosphate buffer (pH 6.5), 13 μL 20 mM GSH, and 13 μL 20 mM CDNB in 95% ethanol.	340 nm	[59]
*Apis mellifera* (honeybee)	Abdomen	Flusilazole (2.3–37 mg/L),	7 days	ns	GST assay kit (Nanjing Jiancheng Bioengineering Institute).	-	[60]
		thiamethoxam (0.3–5.5 μg/L),		ns			
		flusilazole (0.06–0.92 mg/L) + thiamethoxam (0.009–0.014 μg/L)		ns			
*Apis mellifera* (honeybee)	Head Abdomen Midgut	Imidacloprid (0.1–10 μg/L), difenoconazole (0.1–10 μg/L), or glyphosate (0.1–10 μg/L) + binary combination	20 days	Concentration-, tissue-, and time-dependent	GSH-CDNB assay [127]: 1 mM EDTA, 2.5 mM GSH, 1 mM CDNB, and 100 mM disodium phosphate (pH 7.4).	340 nm	[61]
*Apis mellifera* (honeybee)	Midgut	Environmental pollution	-	-	GSH-CDNB assay [125]: 1 mM EDTA, 2.5 mM GSH, 1 mM CDNB as the substrate and 40 mM sodium phosphate (pH 7.4).	340 nm	[62]
*Bombyx mori* (silkworm) larvae	Midgut, Malpighian tubules, and fat body with adhered tissues	Polystyrene nanoplastics (0.5 mg/kg in 0.5 g of feeding)	21 days	ns	GSH-CDNB assay [98]: GSH (20 mM) in 100 mM phosphate buffer (pH 7.4) and the substrate CDNB (20 mM).	340 nm	[65]
*Chironomus riparius* (harlequin fly), larvae	Whole body	Esfenvalerate (0.075–1.2 mg/L)	24 h	ns	GSH-CDNB assay [125]: the activity of GST was determined in post mitochondrial supernatant following conjugation of GSH with CDNB.	340 nm	[20]
*Chironomus kiiensis*, larvae	Whole body	Phenol (1–100 mg/L)	6–48 h	Decreased (100 mg/L)	GSH-CDNB assay [128]: 100 μL of supernatant, 500 μL of 1 mmol L^−1^ GSH, 100 μL of 0.1 mol L^−1^ CDNB and 0.1 mol L^−1^ PBS up to 3.0 mL.	3 min at 340 nm	[43]
			12–48 h	Decreased (10 mg/L)			
			48 h	Decreased (1 mg/L)			
*Chironomus riparius* (harlequin fly), larvae	Whole body	Spinosad (0.5–8 μg/L)	48 h	ns	GSH-CDNB assay [125].	3 min at 340 nm	[44]
		Indoxacarb (2–8 μg/L)		Increased (4 μg/L)			
*Chironomus riparius* (harlequin fly), larvae	Whole body	Tetracycline (1.83–174 μg/L)	48 h	Decreased (18.5 μg/L)	GSH-CDNB assay [129].	340 nm	[45]
*Chironomus riparius* (harlequin fly), larvae	Whole body (protein fraction)	Tire rubber micro particles (1 and 10 mg/L)	24 h	ns	GSH-CDNB assay [125].	-	[46]
		Tire rubber leachate (0.0125 and 5%)		ns			
*Drosophila melanogaster* (fruit fly)	Whole body	CdCl_2_ (0.05 mM)	exposure went through the entire growth cycle, from 1–3 days larvae to hatching adults	Decreased	GSH-CDNB assay [130]: 270 μL of a solution made up of (20 μL of 0.25 M potassium phosphate buffer, pH 7.0, with 2.5 mM EDTA, 10.5 μL of distilled water and 500 μL of 0.1 M GSH at 25 °C), 10 μL of 25 mM CDNB and 20 μL of sample (1:5 dilution).	5 min (10 s intervals) at 340 nm	[47]
		CdCl_2_ (0.05 mM) + hesperidin (0.05 and 0.01 mM)		Decreased (0.05 mM)			
*Drosophila melanogaster* (fruit fly)	Whole body (cytosolic or mitochondrial fraction)	Paraquat (PQ) (10–40 mM)	24 h	Increased (20 mM)	GSH-CDNB assay [131]: 50 μL of sample added to 0.1 M sodium phosphate buffer, pH 6.5, with1 mM EDTA, 20 mM reduced GSH, and 20 mM CDNB.	340 nm	[48]
*Drosophila melanogaster* (fruit fly)	Head	FeSO_4_ (1 and 10 μM)	10 days	ns	GSH-CDNB assay [130]: 270 µL of solution (0.25 M potassium phosphate buffer (pH 7.0) + 2.5 mM EDTA + 0.1 M GSH),10 µL of 25 mM CDNB and 20 µL of sample (1:5 dilution).	5 min (10 s intervals) at 340 nm	[49]
		Rotenone (50 μM)		ns			
		FeSO_4_ (1 and 10 μM) + rotenone (50 μM)		Decreased (1 μM)			
*Drosophila melanogaster* (fruit fly)	Whole body	*Eugenia uniflora* leaves essential oil (3 mg/L)	3, 6, 12 h	Increased (6 h)	GSH-CDNB assay [130]: 100 mM phosphate buffer pH 7.0, 1 mM EDTA, 1 mM GSH, and 2.5 mM CDNB.	340 nm	[50]
*Drosophila melanogaster* (fruit fly)	Adults whole body	Sucrose/yeast larval diet (0.25–20% as sucrose)	up to pupation (96 h)	-	-	.	[51]
*Drosophila melanogaster* (fruit fly) larvae and adults	Whole body	CuSO_4_ (0.1–1.0 mM)	5, 9, 13 days	Modulated	GSH-CDNB assay [125]: 20 μL of GST buffer (0.25 M potassium phosphate buffer, pH 7.0, containing 2.5 mM ethylenediaminetetraacetic acid (EDTA), 60 μL of sample (the total protein content in each well was 60 μg), 10 μL of 100 mM GSH, and 10 μL of 25 mM CDNB.	8 min (30 s intervals) at 340 nm	[52]
	Head, body (thorax and abdomen)			Sex- and concentration-dependent			
*Galleria mellonella* (wax moth), larvae	Midgut	CuO-NPs (size < 50 µm) (10 µg/L)	15 days	ns	GSH-CDNB assay [125].	340 nm	[68]
	Fat body			Increased			
*Galleria mellonella* (wax moth), larvae	Midgut Fat body	Al_2_O_3_-NPs (<50 µm, or <50 nm) (10–100 mg/L in 10 µL volume)	Single injection	NP size-, dose-, and tissue-dependent	GSH-CDNB assay [125].	340 nm	[69]
*Galleria mellonella* (wax moth)	Midgut	CuSO_4_ (10 mg/L)	72 h	Increased	GSH-CDNB assay [125].	340 nm	[70]
		ZnCl_2_ (30 mg/L)		ns			
		CuSO_4_ (10 mg/L) + ZnCl_2_ (30 mg/L)		Decreased			
	Fat body	CuSO_4_ (10 mg/L)		Increased			
		ZnCl_2_ (30 mg/L)		Increased			
		CuSO_4_ (10 mg/L) + ZnCl_2_ (30 mg/L)		Decreased			
*Galleria mellonella* (wax moth)	Hemolymph	Gibberellic acid (GA_3_) (0.05–5 g/kg)	up to a weight target of approx. 18 g	Modulated	GSH-CDNB assay kit (Cayman Chemical) [125].	5 min at 340 nm	[71]
*Galleria mellonella* (wax moth), larvae	Whole body	Juglone (0.25–1.15 mg/g)	up to a weight target of approx. 18 g	Modulated	GSH-CDNB assay kit (Cayman Chemical).	5 min at 340 nm	[72]
*Galleria mellonella* (wax moth), larvae	Hemolymph	Azadirachtin (0.5–3 µg/larva)	24 h delayed analysis	Increased (0.5 µg)	GSH-CDNB assay kit (Cayman Chemical).	5 min at 340 nm	[73]
			72 h delayed analysis	Modulated			
*Hydropsyche pellucidula* larvae	Whole body	Polypropylene microplastics (125 µg/L)	10 days	ns	GSH-CDNB assay [125].	5 min at 340 nm	[81]
*Leptinotarsa decemlineata* (Colorado potato beetle) larvae and adults	Whole body	Glyphosate based herbicide (long-term contaminated-soil growth potatoes)	9 days	Increased ^b^	GST assay kit (CS0410, Sigma-Aldrich): 2 μL of each sample in triplicate and our own reagents: Dulbecco’s phosphate-buffered saline (DPBS), 200 mM GSH, and 100 mM CDNB in ethanol.	340 nm	[40]
*Locusta migratoria* (Locust)	Testicular tissues	Al_2_O_3_ NPs (0.03 mg/g body weight)	single injection	Decreased *	GST was estimated according to a previous method [132].	-	[13]
		Al_2_O_3_ NPs (0.03 mg/g body weight) + PAE (*Periplaneta americana* extract) (0.05 mg/g body weight)		ns			
*Oncopeltus fasciatus* (large milkweed bug)	Whole body	FeSO_4_·7H_2_O (2 or 5 g/L) + Ascorbic acid (2 or 5 g/L)	96 h	ns	GSH-CDNB assay [130]. GST activity was set at 30 °C.	2 min at 340 nm	[54]
*Spodoptera litura* (tobacco worm) larvae	Gut	Fipronil (FP) (20–80 mg/L)	12–72 h	Modulated	GSH-CDNB assay [125]: 3 mL contains 50 µL of 50 mM CDNB, 150 µL of 50 mM GSH and 20 µL of supernatant in sodium phosphate buffer (100 mM, pH 6.5).	10 min at 340 nm	[77]
*Trachyderma hispida* (desert beetle)	Midgut	Textile industry soil (metal contaminants)	-	Decreased ^c^	GSH-CDNB assay [125].	-	[16]

^a^ Analysis of variance (ANOVA) was performed to highlight statistical differences between treatment and control groups at a confidence level of 95% (*p* < 0.05), except when differently noted: ^b^, generalized linear models (GLMs); ^c^, Student’s *t*-test; *, *p* < 0.001; ns, no significant variation.

**Table A8:** Determination of glutathione reductase (GR).

Insect	Sample Type	Exposure		Effect (Significant dose) ^a^	Method	Reading Wavelength	Reference
		Contaminant Type (Dose)	Period				
*Apis mellifera* (honeybee)	Thorax–abdomen (cytosolic fraction)	Environmental pollution	-	-	NADPH oxidation [126]: NaH_2_PO_4_ + Na_2_HPO_4_ 100 mM buffer (pH 7), 1 mM GSSG, and 0.06 mM NADPH.	340 nm	[11]
*Chironomus riparius* (harlequin fly), larvae	Whole body	Spinosad (0.5–8 μg/L), or Indoxacarb (2–8 μg/L)	48 h	ns	NADPH oxidation [134].	1 min at 340 nm	[44]
*Leptinotarsa decemlineata* (Colorado potato beetle) larvae and adults	Whole body	Glyphosate based herbicide (long-term contaminated-soil growth potatoes)	9 days	ns ^b^	GR-assay kit (Sigma-Aldrich): assay buffer (100 mM potassium phosphate buffer + 1 mM EDTA, pH 7.5), 2 mM GSSG, 3 mM DTNB, and 2 mM NADPH.	412 nm	[40]

^a^ Analysis of variance (ANOVA) was performed to highlight statistical differences between treatment and control groups at a confidence level of 95% (*p* < 0.05), except when differently noted: ^b^, Gaussian distribution and identity link function (GLMM); ns, no significant variation.

**Table A9:** Determination of glutathione peroxidase (GPx) activity.

Insect	Sample Type	Exposure		Effect (Significance ^a^)	Method	Reading Wavelength	Reference
		Contaminant Type (Dose)	Period				
*Acheta domesticus* (house cricket)	Hemolymph	Diamond nanoparticles	7 weeks	Increased (200 mg/kg)	Cumene oxide reduction: 0.1 M phosphate buffer with additives (2 mM EDTA, 2 mM NaN_3_), GR (1.6 mg protein mL^−1^), 10 mM GSH, 2.5 mM NADPH, supernatant and 10 µL (15 mM) of cumene oxide.	3 min (with an interval of 15 s) at 340 nm	[78]
	Head			ns			
	Gut			Modulated			
	Gonads			Increased (200 mg/kg)			
	Fat body			Modulated			
*Anaceana globulus* (water scavenger beetle)	Whole body	Heavy metal polluted water (Cu, Zn, Fe, Mn, Pb, Co and Cd)	-	Decreased ^b^	The GSSG produced upon the reduction of an organic hydroperoxide (ROOH) by GPx [136].	340 nm	[39]
*Apis mellifera* (honeybee)	Thorax–abdomen (cytosolic fraction)	Environmental pollution	-	-	NADPH oxidation/H_2_O_2_ [126]: NaH_2_PO_4_ + Na_2_HPO_4_ 100 mM buffer, pH 7.5, 1 mM EDTA, 0.12 mM NADPH, 2 mM GSH, 1 U of GR, 1 mM NaN_3_, and 0.6 mM H_2_O_2_.	340 nm	[11]
*Apis mellifera* (honeybee) queens and foragers at different ages	Hemolymph	Imidacloprid (5 and 200 μg/kg)	1 month	Age-, role-, and concentration-dependent	Pyrogallol method [87,100,105]: 25 μL of the assay mixture: 125 μM phosphate buffer (pH 6.8), 50 μM pyrogallol, 50 μM H_2_O_2_, and 5 μL hemolymph. This was incubated for 5 min at 25 °C, after which the reaction was stopped by adding 5 μL of 5% H_2_SO_4_.	420 nm	[59]
*Chironomus riparius* (harlequin fly), larvae	Whole body	Spinosad (0.5–8 μg/L)	48 h	Increased (8 μg/L)	NADPH oxidation [137] as a result of GR conversion of GSSG to GSH.	3 min at 340 nm	[44]
		Indoxacarb (2–8 μg/L)		Increased (8 μg/L)			
*Drosophila melanogaster* (fruit fly)	Head	Brilliant black PN (azo dye, E151) (1–5 mg/L)	up to development of the larvae	Decreased (5 mg/L *)	NADPH oxidation [138].	340 nm	[19]
*Galleria mellonella* (wax moth), larvae	Midgut or fat body	CuO-NPs (size <50 µm) (10 µg/L)	15 days	ns	NADPH oxidation (pH 7) [135]: GPx activity in the presence of GSSG-reductase, GSH, cumene hydroperoxide as substrate.	5 min at 340 nm	[68]
*Galleria mellonella* (wax moth), larvae	Midgut Fat body	Al_2_O_3_-NPs (<50 µm and <50 nm studies) (10–100 mg/L in 10 µL volume)	single injection, analysis delayed by 48 h	NPs size-, dose-, and tissue-dependent	NADPH oxidation (pH 7) [135].	5 min at 340 nm	[69]
*Galleria mellonella* (wax moth)	Midgut Fat body	CuSO_4_ (10 mg/L) and/or ZnCl_2_ (30 mg/L)	72 h	Decreased in both matrices in every treatment	NADPH oxidation (pH 7) [135].	340 nm	[70]
*Galleria mellonella* (wax moth), larvae	Whole body	Juglone (0.25–1.15 mg/g)	up to a weight target of approx. 18 g	Modulated	NADPH oxidation with GPx assay kit (703102, Cayman Chemical)	5 min at 340 nm	[72]
*Hydropsyche pellucidula* larvae	Whole body	Polypropylene microplastics (125 µg/L)	10 days	ns ^c^	NADPH/H_2_O_2_ [141].	2 min at 340 nm	[81]
*Leptinotarsa decemlineata* (Colorado potato beetle) larvae and adults	Whole body	Glyphosate-based herbicide (long-term contaminated-soil growth potatoes)	9 days	ns ^d^	GPx assay (CGP1, Sigma-Aldrich): 2 mM H_2_O_2_ instead of t-Bu-OOH as a substrate [140].	340 nm	[40]
*Locusta migratoria* (Locust)	Testicular tissues	Al_2_O_3_ NPs (0.03 mg/g body weight)	single injection	Decreased **	H_2_O_2_ consumption and continuous monitoring of GSSG [139].	-	[13]
		Al_2_O_3_ NPs (0.03 mg/g body weight) + *Periplaneta americana* extract (PAE at 0.05 mg/g body weight)		ns *			

^a^ Analysis of variance (ANOVA) was performed to highlight statistical differences between treatment and control groups at a confidence level of 95% (*p* < 0.05), except when differently noted: ^b^, Student’s *t*-test; ^c^, Kruskal–Wallis test; ^d^, Gaussian distribution and identity link function (GLMM); *, *p* < 0.01; **, *p* < 0.001; ns, no significant variation.

**Table A10:** Determination of glutathione (GSH) content.

Insect	Sample Type	Exposure		Effect (Significant Dose/Time) ^a^	Method	Reading Wavelength	Reference
		Contaminant Type (Dose)	Period				
*Anaceana globulus* (water scavenger beetle)	Whole body	Heavy metal polluted water (Cu, Zn, Fe, Mn, Pb, Co and Cd)	-	Decreased ^b^	DTNB reduction with GSH to produce a yellow compound [144].	412 nm	[39]
*Atta sexdens* (leafcutter ant), workers	Fat bodies	Sulfaramid (0.005% in acetone)	24–48 h	ns	GSH-DTNB assay: 50 μL of samples (or standard), 175 μL of 0.3 mM NADPH, and 10 μL of glutathione reductase, incubated for 10 min at 37 °C and then 25 μL of 6 mM DTNB was added.	5 min at 412 nm	[64]
		Fipronil (0.001% in acetone)	24–72 h	ns			
		Mg-1 complex (2 g/L)	48–96 h	Decreased (48 h)			
*Chironomus riparius* (harlequin fly), larvae	Whole body	Esfenvalerate (0.075–1.2 mg/L)	24 h	Decreased (0.075 mg/L)	GSH-DTNB assay: postmitochondrial supernatant fraction + GSH + DTNB in the presence of GR excess [146].	412 nm	[20]
*Drosophila melanogaster* (fruit fly)	Head	Brilliant black PN (azo dye, E151) (1–5 mg/mL)	up to development of the larvae	Decreased (2.5 mg/L **; 5 mg/L ***)	GSH-DTNB assay [145].	412 nm	[19]
*Drosophila melanogaster* (fruit fly), cells	-	Sb(III) (Potassium antimonyl tartrate trihydrate) (1.2 mg/mL)	entire growth cycle, from embryos to hatching adults	Increased **	GSH assay kit (BC1175, Solarbio, Beijing, China).	-	[21]
*Drosophila melanogaster* (fruit fly)	Whole body (cytosolic fraction)	Paraquat (PQ) (10–40 mM)	24 h	Increased (20 mM)	Fluorimetric method employing o-phthalaldehyde (OPT) [150]: an aliquot of cytosol (0.1 M formic acid was added to stabilize reduced GSH in tissue sample and centrifuged at 5200× *g* for10 min) was allowed to react with OPT (1 mg/mL in methanol) at room temperature for 30 min.	Ex/Em = 345/425 nm	[48]
*Leptinotarsa decemlineata* (Colorado potato beetle) larvae and adults	Whole body	Glyphosate based herbicide (long-term contaminated-soil growth potatoes)	9 days	tGSH increased ^c^ GSH:GSSG ns	Total GSH and the ratio of GSH:GSSG were measured with a ThioStar^®^ glutathione fluorescent detection kit (K005-FI, Arbor Assays, Ann Arbor, MI, USA).	Ex/Em = 405/510 nm	[40]
*Periplaneta americana* (American cockroaches)	Whole body	Bendiocarb (0.1 nM)	1 h	Decreased ^d,^*	GSH-DTNB assay [133]: supernatant was added to 2.3 mL of deionized water, 100 mL of 0.3 M EDTA, 300 mL of 0.32 M tris(hydroxymethyl)aminomethane, and 100 mL of 6 mM DTNB. Samples were maintained at 10 °C for 10 min.	412 nm	[37]

^a^ Analysis of variance (ANOVA) was performed to highlight statistical differences between treatment and control groups at a confidence level of 95% (*p* < 0.05), except when differently noted: ^b^, Student’s *t*-test; ^c^, Gaussian distribution and identity link function (GLMM); ^d^, Mann–Whitney test; *, *p* < 0.01; **, *p* < 0.001; ***, *p* < 0.0001; ns, no significant variation.

**Table A11:** Determination of total reduced thiols.

Insect	Sample Type	Exposure		Effect (Significance) ^a^	Method	Reading Wavelength	Reference
		Contaminant Type (Dose)	Period				
*Drosophila melanogaster* (fruit fly)	Whole body	Rotenone (0.05 mM)	7 days	Decreased	Ellman’s reagent method [133]: 170 μL of 0.1 M potassium phosphate buffer (pH 7.4), 20 μL of the sample, and 10 μL of Ellman’s reagent, DTNB. DTNB reacts with free thiol groups, forming a yellow product known as TNB when interacting with thiol groups. The mixture was incubated for 30 min at room temperature.	405 nm	[15]
		Rotenone (0.05 mM) + Syagrus coronata fixed oil (0.2 mg/mL)		ns			
*Drosophila melanogaster* (fruit fly)	Whole body	CdCl_2_ (0.05 mM)	exposure went through the entire growth cycle, from 1–3 days larvae to hatching adults	Decreased	Ellman’s reagent method [133]: 510 μL of 0.1 M phosphate buffer (pH 7.4), 20 μL of sample, 35 μL of 1 mM DTNB and 35 μL of distilled water. The mixture was incubated for 30 min at room temperature.	412 nm	[47]
		CdCl_2_ (0.05 mM) + hesperidin (0.05 and 0.01 mM)		ns			
*Drosophila melanogaster* (fruit fly)	Head	FeSO_4_ (1, and 10 μM)	10 days	ns	Total thiol level was determined in the fly head according to the Ellman method [133]. Nonprotein thiol was measured as described by Jollow et al. [151]. The reaction mixture included 270 µL of 0.1 M phosphate buffer (pH 7.4), 10 µL of sample, and 20 µL of 1 mM DTNB. The mixture was incubated for 30 min at room temperature.	412 nm after 30 min	[49]
		Rotenone (50 μM)		ns			
		FeSO_4_ (1, and 10 μM) + rotenone (50 μM)		Decreased (1 μM)			

^a^ Analysis of variance (ANOVA) was performed to highlight statistical differences between treatment and control groups at a confidence level of 95% (*p* < 0.05); ns, no significant variation.

**Table A12:** Determination of alpha-tocopherol.

Insect	Sample Type	Exposure		Effect (Significance) ^a^	Method	Reading Wavelength	Reference
		Contaminant Type (Dose)	Period				
*Apis mellifera* (honeybee)	Whole body	AlCl_3_ (20–130 mg/L)	10 days	ns ^b^	Reversed-phase HPLC [153]: homogenate (1.5 mL) was mixed with 1 mL of MeOH (0.1% BHT), vortexed for 30 s, and extracted three times with hexane:acetone (50:50) with 4 mL for the first extraction, and 3 mL for the second and third extractions. The α-tocopherol was detected using the chromatographic method.	292 nm	[17]
		CdCl_2_ (0.005–0.030 mg/L)		Increased ^b^ (0.03 mg/L)			
		PbCl_2_ (0.05–0.30 mg/L)		Increased ^b^ (0.05 mg/L *)			
*Apis mellifera* (honeybee)	Whole body	FeCl_2_·4H_2_O (40, 200 mg/kg) with or without CdCl_2_ (0.03 mg/kg),	10 days	ns	Reversed-phase HPLC [153].	292 nm	[63]
		Atrazine (5 ng/bee), and/or glyphosate (5 ng/bee) with or without CdCl_2_ (0.03 mg/kg)					

^a^ Analysis of variance (ANOVA) was performed to highlight statistical differences between treatment and control groups at a confidence level of 95% (*p* < 0.05), except when differently noted: ^b^, generalized linear models (GLMs); *, *p* < 0.01; ns, no significant variation.

**Table A13:** Determination of lipid damage.

Insect	Sample Type	Exposure		Effect (Significance) ^a^	Method	Reading Wavelength	Reference
		Contaminant Type (Dose)	Period				
*Aiolopus thalassinus* (grasshopper), nymph	Brain, thoracic muscles, and gut	Different polluted level sites	-	Increased ^b^	TBARS assay with xylenol orange [165]: reaction mixture, containing 350 μL supernatant, 1750 μL of 1 mM FeSO_4_, 700 μL of 0.25 M H_2_SO_4_, and 700 μL of 1 mM xylenol orange, was incubated in darkness and at room temperature for 3 h; Then, 10 μL of 0.5 mM cumene hydroperoxides were added to the mixture and incubated for 1 h.	580 nm	[79]
*Aiolopus thalassinus* (grasshopper)	Brain, thoracic muscles, and gut	Fertilizer industry surroundings	-	Increased	TBARS assay with xylenol orange [165]: frozen tissue samples were homogenized (1:10, *w*/*v*) in cold 1.1% phosphoric acid. Then, 0.4 mL of homogenate was mixed with 0.4 mL of 1% TBA/50 mM NaOH/0.1 mM BHT solution and 0.2 mL of 7% phosphoric acid (pH 1.6–1.7). Subsequently, samples were heated for 15 min at 100 °C, and then 1.5 mL of butanol was added.	580 nm	[80]
*Anaceana globulus* (water scavenger beetle)	Whole body	Heavy metal polluted water (Cu, Zn, Fe, Mn, Pb, Co and Cd)	-	Increased ^c^	MDA/TBARS assay [155]: acidic medium at 95 °C for 30 min.	534 nm	[39]
*Apis cerana* (honeybee)	Whole body	CdCl_2_ (5.1 mg/L)	14 days	Increased	MDA assay kit (Nanjing Jiancheng Bioengineering Institute).	-	[56]
*Apis mellifera* (honeybee)	Whole body	Al (AlCl_3_) (20–130 mg/L)	10 days	Increased ^d^ (50 mg/L *)	MDA/TBARS [153]: 20 μL of sodium dodecyl sulfate (SDS) (10%), 150 μL of acetic acid (20% pH 3.5), and 150 μL of TBA (0.6% in 0.05 N NaOH), then, incubated at 100 °C for 1 h.	Ex/Em = 532/553 nm	[17]
		Cd (CdCl_2_) (0.005–0.030 mg/L)		ns ^d^			
		Pb (PbCl_2_) (0.05–0.30 mg/L)		ns ^d^			
*Apis mellifera* (honeybee)	Head	Flusilazole (2.3–37 mg/L),	7 days	Increased (37 mg/L)	MDA assay kit (Nanjing Jiancheng Bioengineering Institute).	-	[60]
		thiamethoxam (0.3–5.5 μg/L),		ns			
		flusilazole (0.06–0.92 mg/L) + thiamethoxam (0.009–0.014 μg/L)		Modulated			
*Apis mellifera* (honeybee)	Whole body	FeCl_2_·4H_2_O (40, 200 mg/kg) with or without CdCl_2_ (0.03 mg/kg),	10 days	Concentration- and combination-dependent	MDA/TBARS [153]: in 200 μL bees’ homogenates.	Ex/Em = 532/553 nm	[63]
		Atrazine (5 ng/bee), and/or glyphosate (5 ng/bee) with or without CdCl_2_ (0.03 mg/kg)					
*Bombyx mori* (silkworm) larvae	Midgut, Malpighian tubules, and fat body with adhered tissues	Polystyrene nanoplastics (0.5 mg/kg in 0.5 g of feeding)	21 days	ns	Oxidative damage [155]: 100 mL of each homogenate were placed in glass tubes with 500 mL of 12% (*v*/*v*) TCA in deionized water, 400 mL of 0.6 M Tris HCl and 500 mL of 0.37% (*v*/*v*) TBA in deionized water and incubated 1 h in boiling water. The suspension was then refreshed in ice and centrifuged at 15,000× *g* for 10 min at 20 °C.	535 nm	[65]
*Bombyx mori* (silkworm) Female adults	Ovary tissue	Graphene oxide nanoparticles (GONP) (25 mg/L in 100 µL of culture medium)	24 h	Increased	MDA/TBARS assay kit (Beyotime Biotechnology).	535 nm	[66]
*Chironomus riparius* (harlequin fly), larvae	Whole body	Esfenvalerate (0.075–1.2 mg/L)	24 h	Increased (1.2 mg/L)	MDA/TBARS assay [156]: 4 μL of a 4% solution of the antioxidant compound butylated hydroxytoluene in methanol.	535 nm	[20]
*Chironomus riparius* (harlequin fly), larvae	Whole body	Spinosad (0.5–8 μg/L)	48 h	Increased (2 μg/L)	MDA/TBARS assay [155,156].	535 nm	[44]
		Indoxacarb (2–8 μg/L)		ns			
*Chironomus riparius* (harlequin fly), larvae	Whole body	Tetracycline (1.83–174 μg/L)	48 h	Increased (18.5 μg/L)	MDA/TBARS assay kit (Nanjing Jiancheng Bioengineering Institute) [155].	-	[45]
*Chironomidae, Gomphus*, and *Lestes*	Whole body	Comparison of different rivers on different insect genera	-	-	MDA assay kit (Bioxytech, MDA-586 TM, Oxis Research TM, Portland, USA): 10 µL of probucol + 200 µL of sample or standard + 640 μL of diluted R1 solution (N-methyl-2-phenylindole in acetonitrile) + 150 μL of R2 solution (concentrated hypo chloric acid), incubated at 45 °C for 1 h.	586 nm	[83]
*Drosophila melanogaster* (fruit fly)	Whole body	Rotenone (0.05 mM)	7 days	Increased	MDA/TBARS [155]: 50 μL supernatant from flies treated with and without the specified conditions were mixed with 150 μL 0.1% phosphoric acid and 100 μL TBA 0.6%. The resulting solution was incubated at 100 °C in water bath for 1 h.	405 nm	[15]
		Rotenone (0.05 mM) + Syagrus coronata fixed oil (0.2 mg/mL)		ns			
*Drosophila melanogaster* (fruit fly)	Head	Brilliant black PN (azo dye, E151) (1–5 mg/mL)	up to development of the larvae	ns	MDA/TBARS assay [155].	532 nm	[19]
*Drosophila melanogaster* (fruit fly), cells	-	Sb(III) (Potassium antimonyl tartrate trihydrate) (1.2 mg/mL)	entire growth cycle, from embryos to hatching adults	Increased **	Commercial kit (BC5245, Solarbio, Beijing, China).	-	[21]
*Drosophila melanogaster* (fruit fly)	Whole body	CdCl_2_ (0.05 mM)	exposure went through the entire growth cycle, from 1–3 days larvae to hatching adults	Increased	MDA/TBARS assay [158]: equal volumes of TCA (10%, *w*/*v*) and TBA (0.75%, *w*/*v*) in 0.1 M HCl. 100 μL of supernatant and 200 μL of stock reagent were incubated at 95 °C for 1 h.	532 nm	[47]
		CdCl_2_ (0.05 mM) + hesperidin (0.05 and 0.01 mM)		ns			
*Drosophila melanogaster* (fruit fly)	Whole body cytosolic fraction	Paraquat (PQ) (10–40 mM)	24 h	ns	TBA method [155]: the reaction mixture contained 500 μL cytosol, 1.5 mL acetic acid (pH 3.5, 20%), 1.5 mL of TBA (0.8% *w*/*v*), 200 μL SDS (8% *w*/*v*). The mixture was heated to boiling, and adducts formed were extracted into 3 mL of 1-butanol.	532 nm	[48]
	mitochondrial fraction			Increased (40 mM)			
*Drosophila melanogaster* (fruit fly)	Head	FeSO_4_ (1, and 10 μM)	10 days	ns	MDA/TBARS assay [155]: the reaction mixture contained 5 µL of 10 mM BHT, 100 µL of 0.67% TBA, 300 µL of 1% O-phosphoric acid, 55 µL of distilled water and 40 µL of supernatant. This was followed by an incubation time of 45 min at 90 °C.	535 nm	[49]
		Rotenone (50 μM)		ns			
		FeSO_4_ (1, and 10 μM) + rotenone (50 μM)		Increased (1 µM)			
*Drosophila melanogaster* (fruit fly)	Adult’s whole body	*Eugenia uniflora* leaves essential oil (3 mg/L)	3, 6, 12 h	Increased (6 h)	MDA/TBARS assay [155]: the supernatant was incubated in acetic acid 0.45 M/HCl buffer pH 3.4, containing TBA 0.28%, SDS 1.2%, at 95 °C over 1 h for color development.	532 nm	[50]
*Drosophila melanogaster* (fruit fly)	Adult’s whole body	Sucrose/yeast larval diet (0.25–20% as sucrose)	up to pupation (96 h)	-	TBARS assay with xylenol orange [165].	580 nm	[51]
*Galleria mellonella* (wax moth), larvae	Whole body	Juglone (0.25–1.15 mg/g)	up to a weight target of approx. 18 g	Increased (0.75 mg/g)	MDA/TBARS assay kit (Cayman Chemical): reaction of MDA with TBA at 95 °C.	530 nm	[72]
*Galleria mellonella* (wax moth), larvae	Hemolymph	Azadirachtin (0.5–3 µg/larva)	24 h delayed analysis	Modulated	MDA/TBARS assay kit (Cayman Chemical), incubation at 95 °C.	530 nm	[73]
			72 h delayed analysis	Increased (1.5 µg)			
*Hydropsyche pellucidula* larvae	Whole body	Polypropylene microplastics (125 µg/L)	10 days	Increased ^b^	Lipid peroxidation was tested using 10% of the extracted S9 protein fraction; this colorimetric reaction was conducted using phosphoric acid 1% (*v*/*v*) and TBA 0.6% (*w*/*v*) [168].	Ex/Em = 520–535 nm	[81]
*Leptinotarsa decemlineata* (Colorado potato beetle) larvae and adults	Whole body	Glyphosate-based herbicide (long-term contaminated-soil growth potatoes)	9 days	ns	TBARS assay with xylenol orange [166]: 45 μL sample, 5 μL 10 mM thiamine pyrophosphate (TPP) or methanol, and 950 μL FOX reagent. Cumene hydroperoxide was used as a standard.	570 nm	[40]
*Locusta migratoria* (locust)	Testicular tissues	Al_2_O_3_ NPs (0.03 mg/g body weight)	single injection	Increased **	MDA/TBARS assay [155]: is based on a reaction between TBA and MDA at 95 °C.	532 nm	[13]
		Al_2_O_3_ NPs (0.03 mg/g body weight) + PAE (*Periplaneta americana* extract) (0.05 mg/g body weight)		ns			
*Oncopeltus fasciatus* (large milkweed bug)	Whole body	FeSO_4_·7H_2_O (2 or 5 g/L) + Ascorbic acid (2 or 5 g/L)	96 h	Increased	MDA/TBARS assay [156].	535 nm	[54]
*Oncopeltus fasciatus* (large milkweed bug), two generations; parental exposed, filial not exposed	Whole body	TiO_2_ NPs	5 days (parental)	ns	200 μL sample were boiled with 1 mL of 10% *w*/*v* TCA plus 100 μL of 500 mg/L butylated hydroxytoluene for 30 min and centrifuged at 2500× *g* for 10 min. To duplicate aliquots of the supernatant, an equal volume of 0.375% *w*/*v* TBA with 10% *v*/*v* sulfuric acid was added, and the reaction mixture was boiled again [167].	532 nm	[55]
		Al_2_O_3_ NPs		ns			
		Al_2_O_3_ (bulk form)		ns			
		TiO_2_ NPs	0 days (filial)	ns			
		Al_2_O_3_ NPs		ns			
		Al_2_O_3_ (bulk form)		Increased			
*Ostrinia nubilalis* (European corn borer)	Whole body	Cd(II) (1–100 mg/kg)	28 days	Increased (41.7 mg/kg)	MDA/TBARS assay.	532 nm	[74]
*Periplaneta americana* (American cockroaches)	Whole body	Bendiocarb (0.1 nM)	1 h	ns	MDA/TBARS assay [159,162]: the samples were incubated with 15% TCA and 0.37% TBA in boiling water bath for 20 min. Butylated hydroxytoluene in ethanol was added to the mixture to prevent artifactual lipid peroxidation during the boiling step. After incubation, samples were centrifuged (15 min, 12,000× *g*) and the absorbance of supernatant was measured.	535 nm	[37]
*Reticulitermes speratus* (termite), queens vs. workers comparison	Whole body	UV-B (312 nm, 12.1 W/m^2^)	20 min	ns	MDA/TBARS assay kit (Cayman Chemical). MDA standard or samples were mixed with 50 μL 10% SDS solution (*w*/*v*) and 1 mL color reagent (0.53% TBA, *w*/*v*) in 10% acetic acid solution (*v*/*v*) and 1.5% NaOH solution (*v*/*v*), and incubated for 30 min at 100 °C. Samples were incubated on ice for 10 min to stop the reaction and then centrifuged at 17,000× *g* for 10 min at 25 °C.	532 nm	[38]
*Spodoptera exigua* cells culture	Cells	Camptothecin and derivates (0.1–10 µM)	24 h	Increased (10 µM)	MDA/TBARS assay kit [154]: supernatant with MDA was heated for 15 min at 100 °C.	532 nm	[75]
			48 h	Increased (10 µM)			
*Spodoptera exigua* (beet armyworm), larvae	Whole body	Cd(II) (44 mg/kg of dry feeding) without starvation	200 generations development	ns ^b^	MDA/TBARS assay [160]: 50 µL of sample was mixed with 50 µL of 10% of TCA. Then, the mixture was centrifuged at 12,000× *g* for 10 min at 4 °C. After centrifugation, 70 µL of 1% of TBA was added to 90 µL of the supernatant, and the mixture was vortexed. Then, the samples were heated at 100 °C for 1 h.	532 nm	[76]
		with 1 day starvation		Increased ^b^			
*Spodoptera litura* (tobacco worm) larvae	Gut	Fipronil (FP) (20–80 mg/L)	12 h	Increased (80 mg/L)	MDA/TBARS [155]: 1.9 mL of 0.1 M sodium phosphate buffer (pH 6.5) and 100 µL of supernatant were mixed at pH 6.5 and then incubated at 37 °C for 1 h. After incubation, TCA (10% *v*/*v*) was added and centrifuged for 15 min at 5000 rpm, and the supernatant was collected. The collected supernatant was boiled for 15 min in a water bath.	532 nm	[77]
			24 h	Increased (40 mg/L)			
			48–72 h	Increased (20 mg/L)			
*Stenopsyche marmorata* (caddisfly) larvae	Whole body (except head, tail, and gut)	Environmental pollution: wastewater treatment plants (comparison of spots and seasons)	-	-	Oxidative damage method using TBARS [155,169].	Ex/Em = 530/560 nm	[82]
*Tenebrio molitor* (mealworm larvae)	Whole body	polyethylene (3 g per 100 larvae)	24 h (dose refreshed every 4 days)	Increased	MDA assay kit (Abbkine Scientific Co., Ltd.).	532 nm, 600 nm	[41]
		polystyrene (3 g per 100 larvae		Increased			
		polyvinyl chloride (3 g per 100 larvae)		Increased			
*Trachyderma hispida* (desert beetle)	Midgut	Textile industry soil (metal contaminants)	-	Increased ^c^	MDA/TBARS assay [155].	-	[16]

^a^ Analysis of variance (ANOVA) was performed to highlight statistical differences between treatment and control groups at a confidence level of 95% (*p* < 0.05), except when differently noted: ^b^, Student’s *t*-test; ^c^, Kruskal–Wallis test; ^d^, Gaussian distribution and identity link function (GLMM); *, *p* < 0.01; **, *p* < 0.001; ns, no significant variation.

**Table A14:** Determination of protein damage.

Insect	Sample Type	Exposure		Effect (Significance) ^a^	Method	Reading Wavelength	Reference
		Contaminant Type (Dose)	Period				
*Aiolopus thalassinus* (grass hopper), nymph	Brain, thoracic muscles, and gut	Different polluted level sites	-	Increased ^b^ (except in gut at high level pollution, no significant change)	Tissues were isolated and homogenized in 5 mL of PBS with additives (Triton X-100, CaCl_2_) and centrifuged at 2000× *g* for 10 min at 4 °C. Then, 800 μL supernatant + 800 μL of 30% TCA, followed by incubation (30 min; room temperature) and centrifugation (5000× *g*; 10 min, 4 °C). Protein carbonyl assay was performed on precipitated pellets [80,171].	366 nm	[79]
*Aiolopus thalassinus* (grasshopper), male and female adults	Brain, thoracic muscles, and gut	Fertilizer industry surroundings	-	Increased (different levels, depending on sex)	800 µL supernatant + 800 µL 30% TCA → incubation for 30 min at room temperature and then centrifuged at 5000× *g* for 10 min at 4 °C. The assay of protein carbonyls was conducted on precipitated pellets [171].	.	[80]
*Apis mellifera* (honeybee)	Whole body	Environmental pollution	-	-	Protein carbonyl content assay kit (Merck KGaA, Darmstadt, Germany): bee proteins were solubilized by milling each bee in a 1.5-mL tube with 500 μL of a protein extraction buffer consisting of 20 mM Tris-HCl (pH 8.0), 30 mM NaCl, and 10% glycerol. Subsequently, the sample was centrifuged at 5000× *g* for 12 min and the supernatant treated with the kit.	375 nm	[10]
*Apis mellifera* (honeybee)	Whole body	Al (AlCl_3_) (20–130 mg/L)	10 days	ns ^c^	Protein concentrations were estimated with bovine serum albumin as the standard [173].	-	[17]
		Cd (CdCl_2_) (0.005–0.030 mg/L)		Increased ^c^ (0.01 mg/L; 0.03 mg/L *)			
		Pb (PbCl_2_) (0.05–0.30 mg/L)		ns ^c^			
*Apis mellifera* (honeybee)	Thorax	Se(IV) (0.6–600 μg/L)	2 days	Increased (600 µg/L *)	Protein carbonyl content assay kit (Sigma-Aldrich)	-	[57]
		Se(VI) (0.6–600 μg/L)		Increased (600 µg/L)			
*Apis mellifera* (honeybee)	Midgut	Environmental pollution	-	-	Protein concentrations were estimated with bovine serum albumin as the standard [173].	-	[62]
*Drosophila melanogaster* (fruit fly)	Whole body	CdCl_2_ (0.05 mM)	exposure went through the entire growth cycle, from 1–3 days larvae to hatching adults	Increased	DNPH/TCA assay [171]: 2 mg of protein was incubated with 10 mM DNPH in 2 M HCl (1 mL) for 30 min at room temperature. The sample proteins were precipitated with 10% (*w*/*v*) TCA and recovered by centrifugation for 5 min at 7500× *g*. The protein pellets were washed 3 times with 1 mL of ethanol/ethyl acetate (ratio 1:1, *v*/*v*) to remove free DNPH reagent and redissolved in 1 mL of 6 M guanidine hydrochloride (pH 2.3).	370 nm	[47]
		CdCl_2_ (0.05 mM) + hesperidin (0.05 and 0.01 mM)		ns			
*Drosophila melanogaster* (fruit fly)	Head	FeSO_4_ (1, and 10 μM)	10 days	ns	DNPH/TCA assay [172]: samples were mixed with 20% TCA (ratio 1:1) to precipitate the protein present in the sample. Next, DNPH was added to the resulting mixture to obtain the stable DNP product that was finally suspended in guanidine hydrochloride (6 M).	375 nm	[49]
		Rotenone (50 μM)		ns			
		FeSO_4_ (1, and 10 μM) + rotenone (50 μM)		Increased (1 μM)			
*Drosophila melanogaster* (fruit fly)	Adult’s whole body	Sucrose/yeast larval diet (0.25–20% as sucrose)	up to pupation (96 h)	-	Flies were homogenized in a ratio of 1:10 (*w*/*v*) in 50 mM potassium phosphate buffer containing 0.5 mM EDTA and 0.5 mM phenylmethylsulfonyl fluoride. Amount of protein was assayed in supernatants after centrifugation (13,000× *g*, 15 min, 4 °C) with bovine serum albumin as the standard [173]. Content of carbonyl groups in proteins was measured detecting the amount of DNP formed in the reaction with DNPH [170].	370 nm	[51]
*Hermetia illucens* (black soldier fly), larvae	Gut tissues	Vegetable, fruit, or kitchen wastes + Cd, Fe, Pb, or catechol	7 days	Cadmium and catechol increase protein oxidation in larvae.	DNPH/TCA assay [171]: samples were homogenized in 5 mL of ice-cold phosphate buffer (containing 0.25 mL TCA (0.1% (*w*/*v*), 0.25 mL Triton x-100 (1%)). Then, the samples were centrifuged at 2000× *g* for 10 min at 4 °C. After that, 800 µL supernatant + 200 µL of 10 mM DNPH prepared in 2 M HCl, followed by incubation for 30 min at room temperature and further precipitation with 1 mL of 10% TCA. The pellet was washed four times with an ethanol/ethyl acetate (1:1) mixture and dissolved in 1 mL of sodium phosphate buffer.	366 nm	[53]
*Reticulitermes speratus* (termite)	Whole body	UV-B (312 nm, 12.1 W/m^2^): queens or workers		ns ^d^	Protein carbonyl colorimetric assay kit (Cayman Chemical): termite whole bodies were homogenized in 200 μL ice-cold buffer (20 mM Tris-HCl, 1 mM EDTA, 2% protease inhibitor cocktail (*v*/*v*)). After centrifugation at 16,200× *g* for 10 min at 4 °C, the supernatants were placed into a new tube with DNPH reagent followed by incubation in the dark at room temperature for 1 h. Then, 1 mL of 20% TCA solution (*w*/*v*) was added to the samples before centrifugation at 16,200× *g* for 10 min. The pellets were washed three times with 1 mL of (1:1) ethanol/ethyl acetate mixture.	370 nm	[38]
*Spodoptera exigua* (beet armyworm), cells culture	Cells	Camptothecin and derivates (0.1–10 µM)	24 h	Modulated	Protein carbonyl content assay kit: 100 μL of sample containing 0.5–2.0 mg of protein and 100 μL of DNPH solution, followed by incubation for 10 min at room temperature + 30 μL of the 100% TCA solution was added to the reaction and incubated on ice for 5 min. The reaction solution was centrifuged at 13,000× *g* for 2 min to carefully remove the supernatant. The resulting pellet was added 500 μL of ice-cold acetone placed in a sonication bath for 30 s and then incubated at −20 °C for 5 min. After that, the pellet was centrifuged at 13,000× *g* for 2 min to remove acetone. Guanidine solution (200 μL, 6 M) was added to the pellet and sonicated again briefly.	375 nm	[75]
			48 h	Increased (10 µM)			

^a^ Analysis of variance (ANOVA) was performed to highlight statistical differences between treatment and control groups at a confidence level of 95% (*p* < 0.05), except when differently noted: ^b^, Student’s *t*-test; ^c^, Kruskal–Wallis test; ^d^, Gaussian distribution and identity link function (GLMM); *, *p* < 0.01; ns, no significant variation.

**Table A15:** Determination of DNA damage.

Insect	Sample Type	Exposure		Effect (Significance) ^a^	Method	Reading Wavelength	Reference
		Contaminant Type (Dose)	Period				
*Acheta domesticus* (house cricket)	Hemolymph	Diamond nanoparticles (20 and 200 mg/kg of feeding)	14 weeks	Increased	Comet assay	-	[78]
*Aiolopus thalassinus* (grass hopper), nymph	Brain	Different polluted level sites	-	Increased ^b^	Comet assay [176,177].	-	[79]
	Thoracic muscles			Increased ^b^			
	Gut			Site-dependent			
*Bombyx mori* (silkworm), cells culture	*BmN* cell line	GONPs (25 mg/L in 100 µL)	24 h	ns	Oxidative DNA damage by 8-hydroxy-2 deoxyguanosine (8-OHdG) using an 8-OHdG kit (KeJing Biological Technology Co., Ltd., Beijing, China).	-	[66]
*Chironomus riparius* (harlequin fly), larvae	Whole body	Spinosad (0.5–8 μg/L)	48 h	ns	DNA damage assay [176,180].	Ex/Em = 360/460 nm	[44]
		Indoxacarb (2–8 μg/L)		ns			
*Galleria mellonella* (wax moth), larvae	Hemolymph	Juglone (0.25–1.15 mg/g)	Up to a weight target of approx. 18 g	Increment of all parameters	Comet assay [175].	-	[72]
*Reticulitermes speratus* (termite)	Whole body	UV-B (312 nm, 12.1 W/m^2^): queens or	20 min	ns ^c^	Oxidative DNA damage by 8-OHdG assay kit (colorimetric, Epigentek).	450 nm within 2–15 min	[38]
		workers		Increased ^c^			
*Spodoptera litura* (tobacco worm) larvae	Gut	Fipronil (FP) (20–80 mg/L)	12–72 h	Modulated	The levels of 8-OHdG in the supernatant were determined using a competitive ELISA kit as per the vendor’s protocol (highly sensitive 8-OHdG Check, Japan Institute for control of Aging, Shizuoka, Japan).	-	[77]
*Trachyderma hispida* (desert beetle)	Midgut	Textile industry soil (metal contaminants)	-	Increased ^c^	DNA damage was detected by the comet assay and was performed under alkaline conditions [78,174].	-	[16]

^a^ Analysis of variance (ANOVA) was performed to highlight statistical differences between treatment and control groups at a confidence level of 95% (*p* < 0.05), except when differently noted: ^b^, Kruskal–Wallis test; ^c^, Student’s *t*-test; ns, no significant variation.
